# Neuromodulatory role and therapeutic potential of N^6^-methyladenosine RNA methylation in neurodegenerative diseases

**DOI:** 10.4103/NRR.NRR-D-24-01648

**Published:** 2025-07-05

**Authors:** Jinyu Zhang, Wenjing Ma, Ranxu Liu, Xiaoheng Li, Zengqiang Yuan, Jinbo Cheng

**Affiliations:** 1 Hengyang Medical School, University of South China, Hengyang, Hunan Province, China; 2 Brain Science Center, Beijing Institute of Basic Medical Sciences, Beijing, China; 3 Center on Translational Neuroscience, College of Life & Environmental Science, Minzu University of China, Beijing, China

**Keywords:** Alzheimer’s disease, amyotrophic lateral sclerosis, cell type, m^6^A RNA methylation, methyltransferase-like 3, multiple sclerosis, neurodegeneration, neuroinflammation, Parkinson’s disease, RNA modification, therapeutic strategy

## Abstract

N^6^-methyladenosine RNA methylation, an essential post-transcriptional modification, dynamically regulates RNA metabolism and plays a crucial role in neuronal function. Growing evidence suggests that dysregulated N^6^-methyladenosine modification contributes to the pathogenesis of neurodegenerative diseases, including Alzheimer’s disease, Parkinson’s disease, multiple sclerosis, and amyotrophic lateral sclerosis. However, the precise mechanisms by which N^6^-methyladenosine modification influences these conditions remain unclear. This review summarizes the role of m^6^A modification and its associated regulators in neurodegeneration, focusing on their involvement in key pathological processes. In Alzheimer’s disease, m^6^A modification contributes to synaptic dysfunction, mitochondrial damage, and neuronal apoptosis. Evidence from APP/PS1, 5xFAD, tau transgenic, and *Drosophila* models demonstrates that regulators such as methyltransferase-like 3 and fat mass and obesity-associated protein influence Alzheimer’s disease progression through neuroinflammation, circular RNAs dysregulation, and autophagy-related mechanisms. In Parkinson’s disease, altered N^6^-methyladenosine regulator expression affects dopaminergic neuron survival and stress responses by modulating mRNA stability and autophagy-related lncRNAs. In multiple sclerosis and amyotrophic lateral sclerosis, N^6^-methyladenosine affects immune activation, myelin repair, and the regulation of disease-associated genes such as *TDP-43*. Beyond N^6^-methyladenosine, other RNA methylation modifications—such as m^1^A, m^5^C, m^7^G, uracil, and pseudouridine—are implicated in neurodegenerative diseases through their regulation of mitochondrial function, RNA metabolism, and neuronal stress responses. Additionally, N^6^-methyladenosine exhibits cell type–specific functions: in microglia, it regulates inflammatory activation and phagocytic function; in astrocytes, it modulates metabolic homeostasis and glutamate-associated neurotoxicity; in neurons, it affects synaptic function and neurodegeneration-related gene expression; and in adult neural stem cells, it controls differentiation, neurogenesis, and cognitive plasticity. Recently, several small-molecule inhibitors targeting methyltransferase-like 3 or fat mass and obesity-associated protein have been developed to modulate N^6^-methyladenosine modification, providing new opportunities for disease intervention, with the targeting of N⁶-methyladenosine-related pathways emerging as a promising therapeutic strategy. However, challenges persist in optimizing the specificity and delivery of these therapeutic approaches.

## Introduction

**Figure 1:**
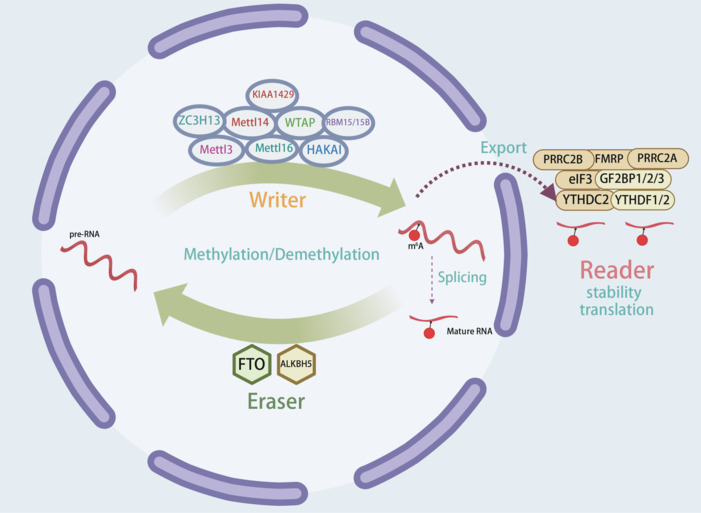
m^6^A RNA methylation modification and its functions. m^6^A RNA methylation is highly dynamic and reversible, regulated through the actions of specialized enzymes: ‘writers,’ ‘erasers,’ and ‘readers.’ Writers such as METTL3, METTL14, WTAP, KIAA1429, RBM15/15B, ZC3H13, and HAKAI mediate the methylation of adenosine residues within RNA. Some erasers such as FTO and ALKBH5 reverse the modification by demethylating its residues. The readers, including proteins such as YTHDF1/2, YTHDC2, eIF3, IGF2BP1/2/3, FMRP, PRRC2A, PRRC2B, and FMRP interpret these modifications and determine the ultimate fate of the RNA. Created with BioRender.com. ALKBH5: AlkB homolog 5; eIF3: eukaryotic translation initiation factor 3; FMRP: fragile X mental retardation protein; FTO: fat mass and obesity-associated protein; HAKAI: E3 ubiquitin-protein ligase Hakai; IGF2BP1/2/3: insulin-like growth factor 2 mRNA-binding protein 1/2/3; KIAA1429: vir-like m^6^A methyltransferase-associated protein; METTL3: methyltransferase-like 3; METTL14: methyltransferase-like 14; pre-RNA: precursor RNA; PRRC2A: proline-rich coiled-coil 2A; PRRC2B: proline-rich coiled-coil 2B; RBM15/15B: RNA-binding motif protein 15/15B; WTAP: wilms tumor 1-associated protein; YTHDC2: YTH domain-containing protein 2; YTHDF1/2: YTH domain-containing family proteins 1/2; ZC3H13: zinc finger CCCH domain-containing protein 13.

N^6^-methyladenosine (m^6^A) RNA methylation is a highly abundant RNA modification, affecting multiple aspects of RNA metabolism, including stability, translation, and splicing (Wang et al., 2014, 2015; Roost et al., 2015; Zhou et al., 2015; Xiao et al., 2016). This modification is catalyzed by methyltransferases (writers), such as methyltransferase-like 3 (METTL3), methyltransferase-like 14 (METTL14), Wilms tumor 1-associated protein (WTAP), KIAA1429, RNA binding motif protein 15/15B (RBM15/15B), and zinc finger CH domain-containing protein 13 (ZC3H13). It is removed by demethylases (erasers), such as fat mass and obesity-associated protein (FTO) and alkB homolog 5 (ALKBH5) (Jia et al., 2011; Zheng et al., 2013; Schwartz et al., 2014; Geula et al., 2015; Patil et al., 2016; Wen et al., 2018; Jiang et al., 2021b), and interpreted by m^6^A-binding proteins (readers) including YTH N^6^-methyladenosine RNA-binding proteins (YTHDF1/2), YTH domain-containing protein 2 (YTHDC2), eukaryotic translation initiation factor 3 (eIF3), insulin-like growth factor 2 mRNA-binding proteins (IGF2BP1/2/3), fragile X mental retardation protein (FMRP), and proline-rich coiled-coil 2A and 2B (PRRC2A and PRRC2B) (Huang et al., 2018; Wu et al., 2019, 2024b; Chen et al., 2023b; Zhang et al., 2024b; **Figure 1**). By mediating the fate of target RNA, m^6^A modification substantially influences cellular homeostasis, developmental processes, and neuronal functions. Recent studies have demonstrated its essential involvement in neurogenesis, synaptic plasticity, and neuronal survival, highlighting its potential implications for neurological diseases (Oerum et al., 2021; Yen and Chen, 2021; Jiang et al., 2022).

Neurodegenerative diseases, including Alzheimer’s disease (AD), Parkinson’s disease (PD), multiple sclerosis (MS), and amyotrophic lateral sclerosis (ALS), are characterized by progressive neuronal dysfunction and loss, often resulting from complex molecular and cellular dysregulation (Heemels, 2016; Dugger and Dickson, 2017; Chi et al., 2018). Extensive research supports the crucial role of m^6^A RNA methylation in the development of these neurodegenerative conditions (Zhang et al., 2022b). In AD, m^6^A RNA methylation regulates amyloid-beta (Aβ) metabolism, Tau phosphorylation, and synaptic function (Huang et al., 2020b; Jiang et al., 2021a; Zhang et al., 2025b). In PD, m^6^A methylation affects α-synuclein (α-syn) aggregation and dopaminergic neuron survival (Geng et al., 2023; Shao et al., 2024). In MS, m^6^A methylation influences immune cell activation and myelin repair (Yamout and Alroughani, 2018; Wu et al., 2019; Zhang et al., 2024b), while in ALS, it modulates TDP-43 pathology and motor neuron degeneration (Martin et al., 2022; McMillan et al., 2023; An et al., 2024). These findings suggest that m^6^A methylation dynamically regulates disease progression and neuronal function through diverse pathways. However, the precise mechanisms by which m^6^A modification exerts these effects remain unclear. Furthermore, the inconsistencies observed in m^6^A patterns across different disease stages and models are indicative of the complexity of its role in neurodegeneration.

This review summarizes the role of m^6^A methylation in neurodegenerative diseases, focusing on its molecular mechanisms, regulatory pathways, and potential clinical applications. We explore the influence of m^6^A methylation dysregulation on disease progression and discuss the therapeutic implications of modulating m^6^A-related pathways. Additionally, we highlight emerging technologies, such as single-cell sequencing and spatial transcriptomics, which may facilitate deeper insights into m^6^A methylation dynamics in nerve cells. By providing a comprehensive understanding of the functions of m^6^A methylation in neurodegeneration, this review aims to establish a theoretical foundation for future disease interventions, particularly with respect to neural regeneration, pathological protein clearance, and neuroinflammation control.

## Search Strategy

We conducted a comprehensive search of PubMed and Web of Science for studies published between 2014 and 2024, using the following search terms in various combinations: m^6^A methylation, METTL3, FTO, ALKBH5, YTHDF1, YTHDF2, YTHDC2, neurodegenerative diseases, Alzheimer’s disease, Parkinson’s disease, multiple sclerosis, amyotrophic lateral sclerosis, N^1^-methyladenosine (m^1^A), 5-methylcytidine (m^5^C), 7-methylguanosine (m^7^G), non-coding RNAs (ncRNAs), long non-coding RNAs (lncRNAs), circular RNAs (circRNAs), microRNAs (miRNAs), microglia, neurons, and neural stem cells (NSCs). Given the growing research on m^6^A modification in neurodegenerative diseases, we prioritized studies providing mechanistic insights. We included peer-reviewed articles in English that presented experimental data on m^6^A methylation in neurodegeneration. Studies were excluded if they lacked experimental findings.

## Relationship Between m^6^A Methylation and the Pathological Processes of Neurodegenerative Diseases

### Alzheimer’s disease

**Figure 2:**
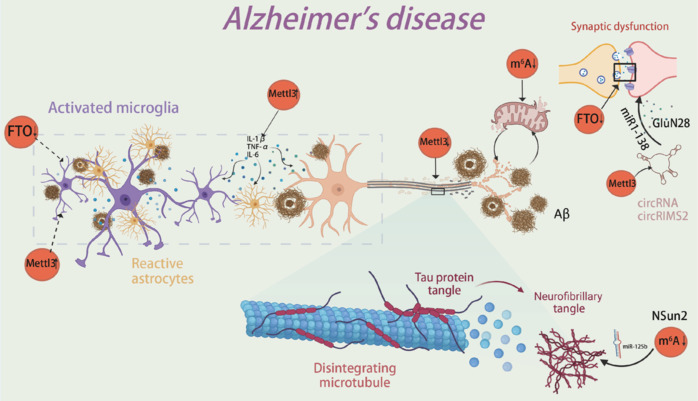
m^6^A RNA methylation and AD. Microglial activation, triggered by Aβ deposition, is a hallmark of AD. While microglia have the ability to clear Aβ plaques and prevent their accumulation, their activation also leads to the release of proinflammatory cytokines (e.g., TNF-α, IL-1β, IL-6), which induce the formation of A1-type reactive astrocytes. This persistent inflammation exacerbates microglial activation and neuronal damage. The upregulation of METTL3 and downregulation of FTO may promote the release of proinflammatory cytokines. Reduced METTL3 levels lead to decreased m^6^A modification in hippocampal neurons, resulting in synaptic loss and cell death. Lower expression of FTO affects the stability of mRNA encoding synaptic proteins, impairing synaptic transmission and structure. Additionally, NSun2-mediated reduction of m^6^A causes dysregulation of miR-125b and hyperphosphorylation of Tau, further accelerating neurotoxicity. METTL3-modified circRIMS2 activates UBE2K-dependent degradation of GluN2B, worsening synaptic dysfunction. Aβ accumulation also damages mitochondria, reducing ATP production and exacerbating neuronal damage. Furthermore, decreased m^6^A weakens mitochondrial function, impairing energy production and making neurons more vulnerable to oxidative stress. Created with BioRender.com. AD: Alzheimer’s disease; Aβ: amyloid-beta; circRIMS2: circular RNA RIMS2; FTO: fat mass and obesity-associated protein; GluN2B: glutamate ionotropic receptor NMDA type subunit 2B; IL-1β: interleukin-1 beta; IL-6: interleukin-6; METTL3: methyltransferase-like 3; miR-125b: microRNA-125b; miR-138: microRNA-138; NSun2: NOP2/Sun RNA methyltransferase 2; TNF-α: tumor necrosis factor-alpha; UBE2K: ubiquitin-conjugating enzyme E2K.

AD is the most common cause of dementia, accounting for 60%–80% of all cases. It has become one of the most costly, deadly, and burdensome diseases, presenting significant challenges to both families and society. It is therefore crucial to understand the pathological mechanisms of the disease and to identify potential treatment strategies (Scheltens et al., 2021; **Figure 2**). A recent study revealed abnormal m^6^A expression in the brains of AD patients (Castro-Hernández et al., 2023), suggesting that m^6^A modification may play a significant role in AD progression. Additionally, as AD involves different pathological mechanisms at various stages, m^6^A levels may fluctuate accordingly, potentially influencing disease progression (**Additional Table 1**).

**Additional Table 1:** Roles of m^6^A modification in early and late stages of AD and AD animal models

AD stage/Model	m^6^A Modification	Role	Reference
Early stage of AD	Decreased METTL3 and WTAP	Aβ clearance affected, and increased Tau phosphorylation	Yin et al., 2023; Gao et al., 2024
	Decreased m^6^A (NSun2-mediated)	miR-125b dysregulation, Tau hyperphosphorylation, and increased neurotoxicity	Zhang et al., 2021; Kim et al., 2023
Late stage of AD	Increased METTL3-mediated m^6^A	Synaptic dysfunction, neuronal apoptosis, and cognitive decline	Wang et al., 2023c
	Decreased m^6^A modification	Mitochondrial dysfunction, oxidative stress, and increased neuronal apoptosis	Swerdlow and Khan, 2004; Kahl et al., 2024
APP/PS1 mouse model	Decreased FTO expression	Synaptic dysfunction, cognitive decline, and neuroinflammation	Engel et al., 2018; Han et al., 2020b
	Increased METTL3 expression	p-Tau clearance, enhanced autophagy, and alleviated AD pathology	Han et al., 2020b; Tang et al., 2023b
	Increased METTL3-mediated m^6^A on circRNA	Synaptic dysfunction, memory impairment, and GluN2B degradation	Mahmoudi and Cairns, 2023; Wang et al., 2023c
5XFAD Mice	Increased METTL3 expression	Enhance neuroinflammation by increasing microglial activity and proinflammatory cytokine expression (TNF-α, IL-13)	Yu et al., 2023; Wu et al., 2024c
	FTO-mediated demethylation	Inhibit neuroinflammation by removing m^6^A modification from inflammatory factors	
	Affect synaptic function	Impair synaptic plasticity and exacerbate cognitive decline by modifying genes involved in neurotransmission	
Tau transgenic mice model	METTL3 upregulation	Promote autophagic degradation of phosphorylated Tau, reduce Tau accumulation and alleviate neurodegeneration and neuroinflammation	Tang et al., 2023b
	m^6^A modification through autophagy-related genes (e.g., *LC3* and *Beclin-1*)	Regulate autophagy-related genes to influence Tau degradation and clear excess Tau	Chen et al., 2021
Drosophila model	METTL3 upregulation	Enhance the translation of neurodevelopmental genes, and improve neuronal function	Sami et al., 2022
	m^6^A modification affecting circMbl	m^6^A-modified circMbl is highly enriched in the *Drosophila* Tau transgenic model, amplifying Tau-induced neurotoxicity	Atrian et al., 2024
	m^6^A modification via METTL3/Ythdf pathway	Regulate behavior and neural function, revealing its selective role in neural regulation	Kan et al., 2021

Aβ: Amyloid-beta; AD: Alzheimer's disease; APP/PS1: amyloid precursor protein/presenilin 1; Beclin-1: BECN1 gene-encoded protein; circMbl: circular RNA muscleblind; circRNA: circular RNA; FTO: fat mass and obesity-associated protein; GluN2B: glutamate ionotropic receptor NMDA type subunit 2B; IL-1β: interleukin-1 beta; LC3: microtubule-associated protein 1A/1 B-l ight chain 3; METTL3: methyltransferase-like 3; m^6^A: N^6^-methyladenosine; NSun2: NOP2/Sun RNA methyltransferase 2; Tau: microtubule-associated protein tau; TNF-α: tumor necrosis factor-alpha; WTAP: wilms tumor 1-associated protein; Ythdf: YTH domain-containing family protein.

#### m^6^A modification in the early stage of Alzheimer’s disease

Cerebrospinal fluid (CSF) biomarkers are commonly used in clinical practice to aid in the diagnosis of AD (Jack et al., 2010; Jack and Holtzman, 2013; Mantzavinos and Alexiou, 2017). Levels of amyloid-β peptide (Aβ), total tau (t-tau), and phospho-tau (p-tau) in CSF have been used as specific clinical biomarkers for AD, because they reflect the pathological processes of Aβ_42_ aggregation and Tau hyperphosphorylation (Dubois et al., 2007). Low Aβ levels appear in the CSF in the early stages of AD; this is used to predict the progression from mild cognitive impairment (MCI) to AD (Buchhave et al., 2012). Similarly, high levels of p-tau and t-tau in the CSF can accurately predict the early stages of AD (Ringman et al., 2008; Fagan et al., 2009). Therefore, in the early stages, Aβ plaque formation and Tau protein hyperphosphorylation play a crucial role in the pathological progression of the disease (Salvadó et al., 2024), disrupting neuronal function and accelerating cell death by triggering inflammatory responses and apoptosis.

m^6^A RNA methylation has been implicated in Aβ metabolism and Tau pathology. WTAP and METTL3 levels are significantly reduced in AD, with METTL3 declining earlier (Gao et al., 2024). Notably, METTL3-mediated m^6^A modification influences Aβ clearance, suggesting it plays a critical role in AD progression (Yin et al., 2023). Additionally, m^6^A modification affects tau toxicity by regulating miR-125b and Tau phosphorylation, with NSun2-mediated m^6^A alterations contributing to neurotoxicity and disease progression (Zhang et al., 2021; Kim et al., 2023). A reduction in m^6^A modification might increase Tau overexpression and hyperphosphorylation, leading to neurofibrillary tangle (NFT) formation in AD.

AD research has frequently made use of APP/PS1 double transgenic mice, which carry mutations in the *APP* and *PSEN1* genes, resulting in excessive Aβ production and rapid amyloid plaque accumulation, especially in the cortex and hippocampus (van Groen et al., 2006; Sasaguri et al., 2017). In this model, by affecting both mRNA and circular RNAs (circRNAs), m^6^A modification has profound effects on synaptic function, neuroinflammation, and cell survival (Zhang et al., 2023c). FTO was significantly reduced in APP/PS1 mice, resulting in an accumulation of m^6^A modification in the mRNA of multiple genes associated with synaptic function and neuroinflammation (Engel et al., 2018; Han et al., 2020b). Decreased FTO expression might impair the stability and translation efficiency of synaptic protein mRNAs, weakening synaptic transmission and inducing degenerative changes in synaptic structure, suggesting the importance of FTO in AD. In addition, METTL3 exhibited an upregulation trend in this mouse model (Han et al., 2020b). An increment of METTL3 promoted the clearance of p-tau by stabilizing STUB1 mRNA and enhancing autophagy, improving AD pathology and cognitive function (Tang et al., 2023b).

Recently, increasing attention has been paid to the involvement of m^6^A modification in circular RNAs (circRNAs) in AD, complementing the traditional m^6^A regulatory pathway in m^6^A epigenetic regulation in AD pathology. circRNAs exhibit high stability and abundance in the nervous system, especially in regions associated with cognition and memory (Mahmoudi and Cairns, 2023). For example, circRIMS2 significantly increased in APP/PS1 mice via METTL3-dependent m^6^A modification, which activated UBE2K and increased the ubiquitination of GluN2B. The ubiquitination of GluN2B accelerated its degradation, triggering synaptic dysfunction and memory impairment. Additionally, inhibition of METTL3 or blocking GluN2B ubiquitination was shown to significantly alleviate synaptic dysfunction, revealing the molecular mechanism underlying the synergistic effect of m^6^A modification in circRIMS2 in AD (Wang et al., 2023c).

#### m^6^A modification in the late stage of Alzheimer’s disease

The pathological features of the advanced stages of AD are more complex than those of the early stages. In addition to increased amyloid plaque deposition, there is significant neuronal loss and synaptic dysfunction, which are closely associated with severe cognitive impairments (Seppälä et al., 2012; Silva et al., 2012; Lloret et al., 2019; Shi et al., 2019; Dubois et al., 2023; Salvadó et al., 2024; Wankhede et al., 2024).

In late stages of AD, synaptic dysfunction is a key factor contributing to the decline in cognitive functions (Majkutewicz et al., 2024). Elevated intracellular calcium levels, associated with Aβ plaques and tau tangles, result in the degradation of synaptic structures, exacerbating memory loss and cognitive deficits (Chakroborty and Stutzmann, 2011). m^6^A modification plays an important role in this process. For example, an increase in m^6^A levels results in a decrease in Lnc-D63785 levels, promoting the accumulation of miR-422a, a molecule linked to cell apoptosis (Xu et al., 2020). Additionally, METTL3 modifies circRIMS2 and activates UBE2K-dependent ubiquitination, contributing to synaptic dysfunction and memory impairment in AD (Wang et al., 2023c). These findings suggest the significant involvement of m^6^A modification in AD progression through regulating neuronal survival and synaptic function. Mitochondria serve as the energy hubs of cells, which are crucial for sustaining neuronal survival (Nunnari and Suomalainen, 2012). Aβ accumulation in the brain can damage mitochondria. For instance, Aβ can reduce the activity of key mitochondrial enzymes, such as cytochrome c oxidase, decreasing oxygen consumption and ATP production and leading to cellular energy deficits and neuronal damage (Du et al., 2011). The expression of genes related to mitochondrial biogenesis and function is disrupted by multiple factors, including post-transcriptional regulatory processes such as m^6^A methylation. Reduced m^6^A levels may weaken mitochondrial generation capacity, resulting in insufficient energy supply and increased vulnerability to oxidative stress (Swerdlow and Khan, 2004; Kahl et al., 2024). The increase in oxidative stress further exacerbates neuronal dysfunction (Butterfield and Halliwell, 2019). Ultimately, the cumulative effects of these metabolic disorders and mitochondrial dysfunction lead to increased neuronal apoptosis and accelerated pathological progression in the advanced stages of AD.

#### Animal models

Animal models have become essential tools for studying disease mechanisms and screening therapeutic targets in AD research, as they can replicate key pathological features of human AD, including Aβ plaque formation, tau protein hyperphosphorylation, synaptic dysfunction, and neuronal degeneration. Different animal models emphasize various pathological features, making them indispensable for investigating specific aspects of AD.

#### 5×FAD mouse models

The 5×FAD mouse is a well-established transgenic model of AD, created by introducing five pathogenic mutations into the mouse genome, two in the human *APP* gene, and three in the presenilin 1 (*PS1*) gene. This mouse model expresses high levels of Aβ and rapidly develops hallmark characteristics of AD, such as Aβ deposition, cognitive impairment, and synaptic loss (Oakley et al., 2006). Neuroinflammation plays a critical role in AD pathology, and 5×FAD mice exhibit a significant neuroinflammatory response. The activation of microglia and astrocytes in response to Aβ plaques further accelerates Aβ deposition and neuronal damage (Crapser et al., 2020; Lecca et al., 2022). In this context, m^6^A modification may influence neuroinflammation by modulating the expression of immune response-associated genes. In 5×FAD mice, m^6^A methylation enhances the neuroinflammatory response by promoting microglial activity and pro-inflammatory cytokine expression. METTL3 overexpression increases the expression of pro-inflammatory genes, including TNF-α and IL-1β, by upregulating m^6^A methylation, thereby exacerbating neuroinflammation and neuronal damage. Conversely, demethylases such as FTO play an inhibitory role in this process by removing m^6^A modifications from inflammatory factors, potentially suppressing the neuroinflammatory response (Yu et al., 2023; Wu et al., 2024c).

*Tau transgenic mouse models*

Hallmark features of AD include abnormal phosphorylation of tau protein and the development of NFTs. Tau protein plays a crucial role in stabilizing microtubule structures in neurons and supporting axonal transport. NFT formation disrupts microtubule stability and function, leading to neuronal death and synaptic loss (Sun et al., 2021). Generally, tau transgenic mice exhibit abnormal tau phosphorylation and cognitive impairments. Tau accumulation also triggers a neuroinflammatory response, exacerbating the pathological progression of AD (Van der Jeugd et al., 2012; Hochgräfe et al., 2013).

Studies using tau transgenic mouse models have reported that m^6^A modification regulates tau expression by influencing the transcription of the MAPT gene, which encodes tau protein. Furthermore, m^6^A modification can indirectly affect tau degradation by regulating genes associated with autophagy and proteasomal degradation, both of which are essential for maintaining cellular protein homeostasis (Wang and Mandelkow, 2016). For instance, METTL3 upregulation promotes the degradation of p-Tau through STUB1-mediated autophagic pathways (Tang et al., 2023b). Additionally, m^6^A modification regulates autophagy-related genes such as *LC3* and *Beclin-1*, influencing autophagic activity and tau clearance (Chen et al., 2021). Therefore, m^6^A modification not only directly influences tau expression but also modulates its degradation pathways.

*Drosophila models*

*Drosophila* melanogaster has emerged as a key model organism for AD research due to its compact genome, short life cycle, and neurological similarity to mammals. *Drosophila* models are primarily used to mimic the key pathological features of AD, including Aβ and tau pathology, mitochondrial dysfunction, oxidative stress, neurodegeneration, and behavioral changes (Jiang and MacNeil, 2023). AD *Drosophila* models include the Aβ model, the AβPP-BACE1 model, and the tau transgenic model (Tsuda and Lim, 2018). The AβPP-BACE1 model expresses human amyloid precursor protein (AβPP) and BACE1, leading to the generation of Aβ peptides and other cleavage products. Neuronal cell death in this model is associated with changes in Aβ_1–42_ levels and alterations in the mRNA expression of lysosomal membrane proteins, such as LAMP1 (Bergkvist et al., 2020). The tau transgenic model expresses the Tau^R406W^ mutant. Pathological tau not only exacerbates the production of reactive oxygen species (ROS) but also increases ethanol sensitivity, suggesting a close relationship between tau pathology, oxidative stress, and behavioral changes, and thus highlighting the crucial role of tau in AD pathogenesis (Nikookar et al., 2021). Additionally, excessive mitochondrial elongation and increased mitochondrial fission contribute to the exacerbation of neurodegenerative lesions (Jiang and MacNeil, 2023).

The m^6^A modification is highly prevalent in the brains of *Drosophila* melanogaster, where it plays a crucial role in regulating aging and brain stress. Research investigating the effects of blue light exposure (BLE) on *Drosophila* aging found that m^6^A methylation sites were enriched in the 5′ untranslated regions (UTRs) and specifically regulated the expression of aging-related genes, such as Tor and circadian rhythm genes (Huang et al., 2023). Furthermore, m^6^A also influences mRNA decay in neuroblasts and neurons within the nervous system. For example, METTL3 enhances the neurodevelopmental gene translation via m^6^A modification, rather than through the regulation of mRNA stability, ultimately improving neuronal function (Sami et al., 2022). Additionally, tau pathology is involved in regulating m^6^A-modified circular RNA (circRNA). The m^6^A-modified circMbl was found to be highly enriched in the *Drosophila* tau transgenic model, exacerbating tau-induced neurotoxicity (Atrian et al., 2024). Furthermore, m^6^A regulates behavior and neural function in *Drosophila* via the METTL3/Ythdf pathway (Kan et al., 2021). Collectively, these findings highlight the *Drosophila* model as a valuable experimental tool for investigating the role of m^6^A in the pathogenesis of AD.

### Parkinson’s disease

PD is a neurological disorder characterized by the progressive degeneration of dopaminergic neurons and the abnormal formation of α-syn (Tang et al., 2025). While motor symptoms are the hallmark of PD, cognitive deficits and psychiatric disturbances often manifest as the disease progresses (Kalia and Lang, 2015). PD’s core pathological features include α-syn aggregation, mitochondrial dysfunction, and neuroinflammation.

The expression patterns of m^6^A-related genes in PD samples differ significantly from those in healthy controls. Specifically, the levels of METTL3, YTHDC1, HNRNPC, HNRPA2B1, IGFBP1, IGFBP3, and ELAVL1 were increased in PD samples, while YTHDC2 and LRPPRC were decreased (Yan et al., 2024). These changes may be linked to the pathogenesis of PD. In a 1-methyl-4-phenyl-1,2,3,6-tetrahydropyridine (MPTP)-induced PD mouse model, an association was found between dysregulated levels of m^6^A regulators and motor dysfunction and cognitive deficits (Yu et al., 2022). Additionally, METTL3 overexpression enhanced the mRNA stability of glutaredoxin (GLRX) and alleviated neuronal damage (Gong et al., 2024). It was reported that FTO promoted dopaminergic neuronal death through m^6^A-dependent regulation of the mRNA stability of ATM (Geng et al., 2023). These findings collectively highlight the critical role of m^6^A modification in regulating neuronal survival and function, underscoring its potential as a therapeutic target for PD treatment.

**Figure 3:**
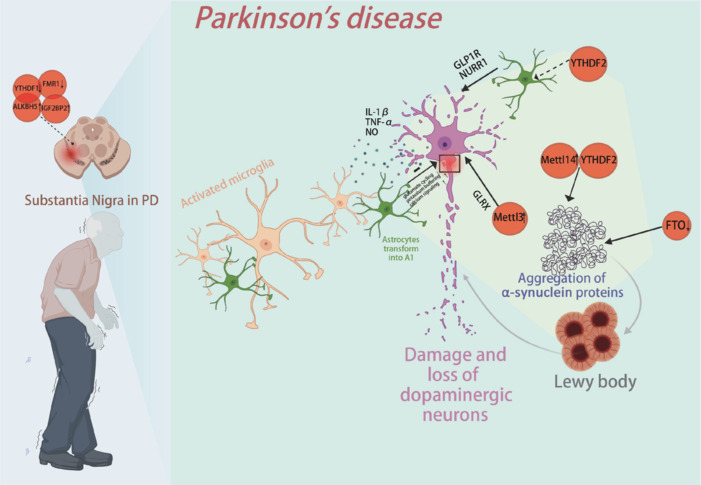
m^6^A RNA methylation and PD. PD is a neurological disorder that impairs motor control and is characterized by the degeneration of dopaminergic neurons and the abnormal accumulation of α-syn. In the substantia nigra, m^6^A regulators such as ALKBH5 and IGF2BP2 are upregulated, while YTHDF1 and FMR1 are downregulated. METTL3 promotes m^6^A modification, stabilizes GLRX mRNA, and provides protection against degeneration in PD mouse models. Overexpression of METTL14 increases m^6^A levels on α-syn mRNA, leading to reduced stability and altered expression of α-syn. YTHDF2 regulates the translation and toxicity of m^6^A-modified α-syn, while knockout of FTO inhibits the upregulation of α-syn and reduces neuronal death in PD models. As PD progresses, the accumulation of α-syn activates microglia, triggering neurotoxic responses, promoting the formation of A1-type astrocytes, and releasing pro-inflammatory factors such as IL-1β, TNF-α, and nitric oxide, which exacerbate neuronal damage. Reactive astrocytes can harm dopaminergic neurons but may also provide protection by secreting neuroprotective molecules like GLP1R and NURR1. Furthermore, YTHDF2 may contribute to neurodegeneration by promoting inflammation through astrocytes. Created with BioRender.com. ALKBH5: AlkB homolog 5; FMR1: fragile X messenger ribonucleoprotein 1; FTO: fat mass and obesity-associated protein; GLP1R: glucagon-like peptide 1 receptor; GLRX: glutaredoxin; IGF2BP2: insulin-like growth factor 2 mRNA-binding protein 2; IL-1β: interleukin-1 beta; METTL3: methyltransferase-like 3; METTL14: methyltransferase-like 14; NO: nitric oxide; NURR1: nuclear receptor related 1 protein; TNF-α: tumor necrosis factor-alpha; YTHDF1: YTH domain-containing family protein 1; YTHDF2: YTH domain-containing family protein 2: α-syn: α-synuclein.

Many noncoding RNAs (ncRNAs) have been found to play crucial roles in apoptosis, α-syn misfolding and aggregation, mitochondrial dysfunction, autophagy, and neuroinflammation during the pathogenesis of PD. This includes microRNAs (Grossi et al., 2021), long noncoding RNAs (lncRNAs) (Fan et al., 2019), and circular RNAs (circRNAs) (Ravanidis et al., 2021). Although the role of m^6^A modification of noncoding RNAs in PD remains unclear, a recent study demonstrated that long-term, low-dose exposure to paraquat (PQ) can lead to abnormal lncRNA expression in neuroblastoma cells (Neuro-2a). Following PQ treatment, m^6^A modification altered lncRNA expression patterns, and treatment with N-acetyl-L-cysteine (NAC)—a ROS scavenger—could partially reverse these m^6^A modifications. The study also identified two m^6^A-modified lncRNAs—CDC5L and STAT3—that influence autophagy-related functions and may play a role in PD (Su et al., 2022). These findings suggest that the interaction between m^6^A modification and lncRNAs may be significant in PD (**Figure 3**).

### Multiple sclerosis

MS is an autoimmune disease in which the immune system erroneously attacks and destroys myelin within the CNS (Göttle et al., 2024; Weerasinghe-Mudiyanselag et al., 2024). This results in myelin loss, neuronal impairment, and various neurological symptoms, including motor, sensory, and cognitive deficits (Doshi and Chataway, 2016). Although the exact cause of MS remains unclear, research suggests that m^6^A methylation may play a role in its progression.

The immune system plays an essential role in MS, with T cells and B cells serving as primary effectors that target myelin (Yamout and Alroughani, 2018). One study found that m^6^A modification significantly affects the immune system by regulating the proliferation, differentiation, activation, and migration of T cells and B cells (Gan et al., 2023). The expression of m^6^A methyltransferases, including METTL3 and METTL14, is closely associated with T cell activation. Alterations in m^6^A modification may lead to abnormal T cell activation in MS. Increased METTL3 expression promotes T cell proliferation (Li et al., 2022b), while decreased METTL14 expression impairs T cell responsiveness to specific antigens (Liu et al., 2023b). Additionally, B cells play a critical role in the immune response in MS and contribute to its chronic progression (Comi et al., 2021). m^6^A modification (e.g., Mettl14 and RMB39B) modulates antibody production and influences B cell activation (Wang et al., 2023b). By regulating genes involved in B cell activation, m^6^A modification may enhance the role of B cells in immune-mediated attacks, leading to myelin damage and neuronal degeneration.

In MS, myelin repair and regeneration are crucial for nerve recovery—even partial restoration of myelin can help improve some functional deficits (Schäffner et al., 2023). m^6^A RNA modification plays an essential role in this regenerative mechanism, particularly in regulating the activity of neuroglial cells, including oligodendrocytes and astrocytes. Our previous research demonstrated that PRRC2A acts as an m^6^A reader in the nervous system, especially during oligodendrocyte development, and regulates the proliferation and myelination of oligodendrocyte progenitor cells (OPCs) (Wu et al., 2019). Furthermore, m^6^A modification mediated by PRRC2B regulates OPC proliferation and differentiation by controlling the expression of key genes related to myelin production (Zhang et al., 2024b). Taken together, these findings suggest the involvement of m^6^A levels in converting OPCs into mature oligodendrocytes, which is a critical step in restoring myelin integrity and function.

**Figure 4:**
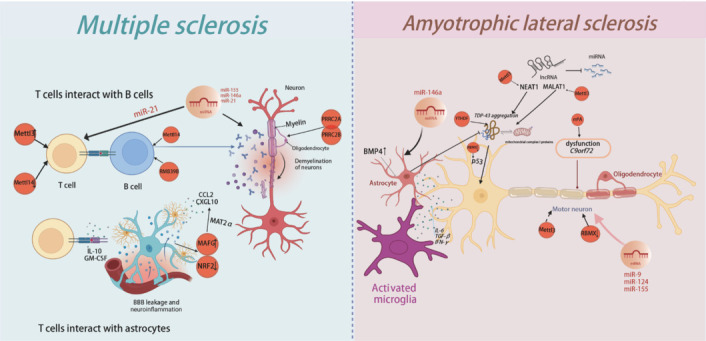
m^6^A RNA methylation in MS and ALS. MS is an autoimmune disease characterized by the immune system’s attack on myelin in the central nervous system, leading to neuronal damage. T and B cells play crucial roles in this process, with abnormal m⁶A modification afffecting T cell activation in MS. Increased levels of METTL3 promote T cell proliferation, whereas reduced levels of METTL14 impair T cell responses. Both METTL14 and RMB39B are involved in regulating B cell activation and antibody production. Additionally, PRRC2A and PRRC2B control the differentiation of oligodendrocyte precursor cells into mature oligodendrocytes, a process influenced by m^6^A levels. MicroRNAs such as miR-155 and miR-146a, modulate neuroinflammation, while miR-21 limits T cell function and immune attacks. METTL3 also regulates miRNA stability, which affects immune responses and contributes to neural damage. Damage to the BBB allows immune cells to invade the CNS and activate astrocytes, which in turn secrete chemokines such as CCL2 and CXCL10, exacerbating inflammation. Reactive astrocytes exhibit altered expression of NRF2 and MAFG, which promotes pro-inflammatory responses. T cells influence astrocyte activity through the release of cytokines such as IL-10 and GM-CSF. ALS is a neurodegenerative disease characterized by motor neuron loss and muscle weakness. Accumulation of TDP-43 leads to mitochondrial dysfunction, binds to mitochondrial mRNA, inhibits the expression of complex I, and accelerates neuronal death. TDP-43 also activates p53, contributing to neuronal damage. m^6^A modification enhances TDP-43 translation and neurotoxicity through YTHDF proteins, while increased m^6^A levels can reduce the accumulation of C9ORF72 repeat RNA. lncRNAs such as NEAT1 and MALAT1 regulate TDP-43 aggregation and ALS-related pathways. The m^6^A modification of these lncRNAs by METTL3 stabilizes them, which enhances neuroprotection. MicroRNAs such as miR-9, miR-155, and miR-124, are critical for neuronal survival. Microglia can exacerbate damage by releasing pro-inflammatory cytokines such as TNF-α and IL-1β. Regulating miR-146a in astrocytes can restore their function and improve motor neuron dynamics. BMP4 promotes astrocyte activation, and inhibiting its signaling pathway can reduce activation and improve motor function. Ultimately, astrocytes protect neurons by limiting TDP-43 aggregation and secreting neurotrophic factors. Created with BioRender.com. ALS: Amyotrophic lateral sclerosis; BBB: blood–brain barrier; BMP4: bone morphogenetic protein 4; C9orf72: chromosome 9 open reading frame 72; CCL2: C–C motif chemokine ligand 2; CXCL10: C–X–C motif chemokine ligand 10; GM-CSF: granulocyte-macrophage colony-stimulating factor; IFN-γ: interferon-gamma; IL-10: interleukin-10; IL-1β: interleukin-1 beta; IL-6: interleukin-6; lncRNAs: long non-coding RNAs; m^6^A: N^6^-methyladenosine; MAFG: musculoaponeurotic fibrosarcoma oncogene homolog G; MALAT1: metastasis-associated lung adenocarcinoma transcript 1; MAT2α: methionine adenosyltransferase 2 alpha; METTL14: methyltransferase-like 14; METTL3: methyltransferase-like 3; miR-124: microRNA-124; miR-146a: microRNA-146a; miR-155: microRNA-155; miR-21: microRNA-21; miR-9: microRNA-9; MS: multiple sclerosis; NEAT1: nuclear paraspeckle assembly transcript 1; NRF2: nuclear factor erythroid 2-related factor 2; p53: tumor protein p53; PRRC2A: proline-rich coiled-coil 2A; PRRC2B: proline-rich coiled-coil 2B; RBM39B: RNA-binding motif protein 39B; RBMX: RNA-binding motif protein X-linked; TDP-43: TAR DNA-binding protein 43; TGF-β: transforming growth factor beta; TNF-α: tumor necrosis factor-alpha; YTHDF: YTH domain-containing family protein.

There have also been significant findings regarding noncoding RNAs (ncRNAs) in MS. miRNAs play a critical role in MS by regulating immune cell function, inflammation, and nerve regeneration (Juźwik et al., 2019). Notably, miR-155 promotes neuroinflammation by activating immune cells (Essandoh et al., 2016), while miR-146a modulates immune responses by suppressing inflammation-related genes (Ma et al., 2014). In contrast, MiR-21 reduces immune attacks by inhibiting T cell function (Fedeli et al., 2021). Importantly, m^6^A modification (e.g., METTL3) can regulate miRNA stability and translation, thereby influencing immune responses and neurological damage (Alarcón et al., 2015; Feng et al., 2023; **Figure 4**).

### Amyotrophic lateral sclerosis

ALS is a degenerative neurological disorder marked by the progressive loss of motor neurons, leading to muscle weakness, atrophy, and respiratory failure. Currently, there are no effective treatments available to slow or halt its progression (Feldman et al., 2022). Research has demonstrated that m^6^A methylation is a critical factor in the development of ALS, playing a role in maintaining RNA homeostasis, controlling neuronal apoptosis, and regulating ALS-related genes such as TDP-43 and C9orf72 (Saberi et al., 2015).

Recent research has suggested that m^6^A dysregulation affects immune cell migration and alters intercellular communication between neurons and glial cells (He et al., 2024a). Research has demonstrated that TDP-43, an important RNA-binding protein in ALS, recognizes m^6^A-modified RNA. This modification plays a crucial role in the binding and function of TDP-43. Additionally, m^6^A modification promotes TDP-43 translation and exacerbates its neurotoxicity through YTHDF proteins (McMillan et al., 2023). In cases of ALS caused by C9orf72 expansion repeats, m^6^A downregulation was associated with increased mRNA stability and elevated gene expression, particularly for genes involved in synaptic activity and neuronal function (Park et al., 2023). It has been reported that increasing m^6^A methylation levels could reduce the accumulation of C9orf72 repeat RNA, thereby improving the survival rate of induced pluripotent stem cell-derived neurons (Li et al., 2023c). Mouse models carrying human superoxide dismutase 1 (hSOD1) mutations, such as G93A and G37R, are widely used in ALS research to mimic motor neuron degeneration and muscle atrophy. Studies have demonstrated significantly elevated m^6^A methylation levels in the spinal cord tissues of hSOD1-G93A mouse models, characterized by decreased expression of the demethylase FTO and increased expression of the methyltransferase METTL3 (Martin et al., 2022; An et al., 2024).

Neuronal apoptosis is a defining characteristic of ALS progression, and m^6^A modification is pivotal in regulating neuronal survival through its effects on apoptosis-related genes. For instance, loss-of-function variants in RNA binding motif protein X-linked (RBMX), an m^6^A methylation-associated protein, have been reported to induce neuronal defects in ALS, specifically affecting excitatory neurons (He et al., 2024b; **Figure 4**). Significant findings have also been reported regarding non-coding RNA (ncRNA) in the context of ALS. NEAT1 has been shown to influence neuroinflammatory pathways and cell death by forming paraspeckles and interacting with RNA-binding proteins, including TDP-43, which is a critical factor in ALS pathogenesis (Tollervey et al., 2011; An et al., 2018). NEAT1 is involved in TDP-43 aggregation and regulates neuron-specific pathways essential for ALS progression (Nishimoto et al., 2013). Additionally, MALAT1 functions as a miRNA sponge in ALS, regulating gene expression and influencing genes involved in apoptosis and DNA repair mechanisms, particularly the *ATM* gene during DNA damage responses (Liu et al., 2021). m^6^A modification has been shown to regulate the stability and function of both NEAT1 and MALAT1, potentially impacting ALS-related biological processes. For example, in astrocytes treated with oxygen-glucose deprivation/reperfusion (OGD/R), METTL3 enhanced NEAT1 stability by promoting its m^6^A modification. NEAT1 functioned as a competing endogenous RNA (ceRNA), binding to miR-377-3p to inhibit its suppression of Nampt, thereby playing a neuroprotective role (Hu et al., 2024). Similarly, METTL3 promoted the m^6^A modification of MALAT1, increasing its expression to further regulate SFRP2 methylation and reduce its expression. This process ultimately alleviated autism-like symptoms and decreased hippocampal neuronal apoptosis (Ming et al., 2022).

## Other RNA Modifications in Neurodegenerative Diseases

Recently, RNA modifications have garnered significant attention within the context of neurodegenerative diseases. As we gain a deeper understanding of these modification mechanisms, it has become increasingly evident that alterations in m^1^A, m^5^C, and m^7^G are closely associated with diseases such as AD, PD, MS, and ALS. These modifications are believed to influence the onset, progression, and clinical symptoms of these disorders. Subsequent sections will explore the specific changes in RNA modifications observed in these diseases and the potential mechanisms involved (**Additional Table 2**).

**Additional Table 2:** Other RNA modifications in neurodegenerative diseases

RNA modification	Disease	Role	Reference
m^1^A	AD	Stabilize neuronal transcripts under oxygen and glucose deprivation/reoxygenation, and protect cells in response to stress. Affect mitochondrial ND5 mRNA, inhibit complex I activity, lead to increased oxidative stress and disrupted energy metabolism, and exacerbate AD pathology.	Qi et al., 2023; Jörg et al., 2024
	PD	Enhance the phase-separation behavior of TDP-43, and exacerbate neuronal damage. Regulate neuronal gene expression and cellular adaptation, and influence neuronal activity and survival under stress conditions. Contribute to the progression of neuronal degeneration and disease.	Qj et al., 2023; Hashmi et al., 2024; Yuan et al., 2024
	ALS	Affect RNA stability and translation efficiency, exacerbate TDP-43 aggregation and toxicity, impair neuronal survival and function, and drive ALS progression.	Zhang et al., 2023a, b; Wang et al., 2024a; Yuan et al., 2024
	MS	Regulate transcription and translation in immune cells, influence neuroinflammation, and promote demyelination and neurological dysfunction.	Correale et al., 2019; Liu et al., 2022; Wu et al., 2022; Zhou et al., 2023
m^5^C	AD	Affect the expression of genes related to synaptic function, neuronal survival, and metabolism, and potentially disrupt normal neuronal function. Catalyzed by RNA methyltransferases such as NSUN6 and NSUN7, alterations in these enzymes' levels may affect the stability and translation efficiency of AD-related mRNA, exacerbating neurodegenerative damage. May function as part of the host immune defense, affect the stability of mRNA involved in inflammatory pathways and modulate the intensity and duration of neuroinflammation.	PerezGrovas-Saltijeral et al., 2023
	PD	Regulate gene expression and post-transcriptional control. Inhibit neuronal mRNA production, disrupt neuronal signaling pathways and potentially influence PD progression.	Piergiorge et al., 2024 Satterlee et al., 2014
m^7^G	AD	Affect AD molecular subtype classification, regulate immune cell characteristics, and offer potential for predicting and classifying AD subtypes based on m^7^G modification.	Ma et al., 2023
		Mettll-driven m^7^G modification enhances the stability and translational efficiency of Sptbn2 mRNA, promoting neuronal generation and development, improving synaptic function, and mitigating neurodegenerative damage.	Li et al., 2023a
		Regulate immune-related genes, affect immune cell infiltration patterns, modulate the intensity and duration of inflammatory responses, and potentially influence AD progression.	Lian et al., 2024

AD: Alzheimer's disease; ALS: amyotrophic lateral sclerosis; ceRNA: competing endogenous RNA; METTL1: methyltransferase-like 1; m^2^A: N^2^-methyladenosine; m^5^C: 5-methylcytosine; m^7^G: 7-methylguanosine; MS: multiple sclerosis; NSUN6: NOP2/Sun RNA methyltransferase 6; NSUN7: NOP2/Sun RNA methyltransferase 7; PD: Parkinson's disease; Sptbn2: spectrin beta chain, non-erythrocytic 2; TDP-43: TAR DNA-binding protein 43.

### N^1^-Methyladenosine

Initially discovered in eukaryotic mRNAs, the N¹-methyladenosine (m^1^A) RNA modification is now recognized as widespread across various cell types, serving an essential function in post-transcriptional regulation (Jin et al., 2022). While m^1^A modification typically occurs at adenosine (A) residues within mRNAs—especially in the 5′ untranslated region (UTR), intronic regions, and areas involved in post-transcriptional splicing—it can also be present in coding regions and the 3′ UTR (Dominissini et al., 2016; Li et al., 2016).

#### N^1^-Methyladenosine modification in Alzheimer’s disease

Recent research highlights the significance of m^1^A modification in AD pathology. This modification influences RNA stability and translational activity in neurons, crucially influencing responses to oxidative stress and maintaining mitochondrial function. Bioinformatics analyses have revealed widespread m^1^A modifications in AD-related genes. Alterations in m^1^A levels within certain genes disrupt gene networks essential for AD progression, exacerbating disease pathology (Tan et al., 2024). Furthermore, the effect of m^1^A on neuronal transcription regulation is heightened under specific stress conditions; for example, research has shown that oxygen and glucose deprivation followed by reoxygenation increases m^1^A modification, which stabilizes neuronal transcripts (Qi et al., 2023). Additionally, m^1^A modification is closely linked to mitochondrial dysfunction in AD. Specifically, m^1^A modifications on mitochondrial ND5 mRNA impair the activity of mitochondrial complex I, leading to elevated oxidative stress and disrupted energy metabolism, further exacerbating AD pathology (Jörg et al., 2024).

#### N^1^-Methyladenosine modification in Parkinson’s disease

Yuan et al. (2024) reported a close association between m^1^A modification and the abnormal aggregation of TDP-43 protein. Their findings showed that expansion of CAG repeats altered the phase-separation of *TDP-43* by enhancing m^1^A modification and exacerbating neuronal damage, suggesting that m^1^A plays a critical role in PD pathogenesis. m^1^A modification also regulates neuronal gene expression and contributes to cellular adaptation mechanisms. Under oxygen and glucose deprivation, m^1^A modifications influenced neuronal activity and survival by enhancing neuronal resilience. Given that oxidative damage is a central pathological feature of PD, the involvement of m^1^A in stress regulation and neuronal function might drive disease progression (Qi et al., 2023). In addition, abnormal m^1^A modification can impair neuronal function, resulting in degeneration (Hashmi et al., 2024). Although research on m^1^A in PD is limited, the findings mentioned above provide a new perspective for understanding the pathological processes underlying PD.

#### N^1^-methyladenosine in multiple sclerosis and amyotrophic lateral sclerosis

The role of m^1^A modification in MS remains unclear; however, evidence suggests it is relevant to immune cell function. m^1^A modifications may influence the onset and progression of neuroinflammation by regulating transcriptional and translational processes in immune cells (Zhou et al., 2023), including T cells (Liu et al., 2022) and macrophages (Wu et al., 2022). Given that abnormal immune activation is a primary contributor to myelin damage and neurological dysfunction in MS (Correale et al., 2019), further research is required to clarify the role of m^1^A modification in this disease. In ALS, m^1^A levels demonstrate an overall downward trend, which may impair RNA stability and translation efficiency, particularly for ALS-related genes such as *TDP-43*. This reduction could exacerbate the neurodegenerative process, suggesting that m^1^A deficiency intensifies the toxic effects of *TDP-43* by altering its stability and promoting aggregation (Yuan et al., 2024). Additionally, early disruption of m^1^A has been shown to impair neuronal survival and function (Zhang et al., 2023a, b; Wang et al., 2024a), potentially influencing ALS onset and progression.

### 5-Methylcytidine

The 5-methylcytidine (m^5^C) modification is a well-established epigenetic mark that is found extensively in both DNA and RNA, including mRNAs, tRNAs, and rRNAs (Bohnsack et al., 2019). m^5^C modification controls gene expression at multiple levels, including by influencing RNA structure and interactions (Trixl and Lusser, 2019), making it a promising therapeutic target in neurodegenerative disease research (Wu et al., 2024a).

#### 5-Methylcytidine modification in Alzheimer’s disease

Emerging evidence suggests that the m^5^C RNA modification may be a key regulator in AD. Bioinformatics analyses have revealed the widespread distribution of m^5^C modifications in several important AD-related genes. The abnormal expression of m^5^C in genes associated with synaptic function, neuronal survival, and metabolism may disrupt normal neuronal function in AD patients (Tan et al., 2024). m^5^C modification is catalyzed by RNA methyltransferases, such as NSUN6 and NSUN7. One study observed substantial changes in the levels of these enzymes in individuals with AD, indicating the potential pivotal role of m^5^C modification in the progression of the disease. Alterations in NSUN6 and NSUN7 levels may influence the stability and translation efficiency of AD-related mRNA, reducing the capacity of neurons to adapt to metabolic stress and thus exacerbating neurodegeneration (PerezGrovas-Saltijeral et al., 2023). Another study explored the link between m^5^C modification and the infectious hypothesis of AD, which suggests that AD onset may be linked to the presence of certain infectious agents, with m^5^C modification potentially serving as a component of the host immune defense mechanism against infection (Teng et al., 2024).

#### 5-Methylcytidine modification in Parkinson’s disease

Recent research suggests that m^5^C modification may be involved in PD pathogenesis. The RNA-binding protein Aly/REF export factor (ALYREF), which binds to m^5^C modifications, is linked to the regulation of post-transcriptional dysregulation in PD (Piergiorge et al., 2024). Furthermore, m^5^C modification has been shown to inhibit the production of neuronal mRNA, thereby disrupting signaling pathways involved in neuronal development. These findings indicate the involvement of m^5^C in the progression of PD (Satterlee et al., 2014).

### 7-Methylguanosine

7-Methylguanosine (m^7^G), a crucial RNA modification located at the 5′ end of mRNAs (Xia et al., 2023), plays a vital role in post-transcriptional RNA regulation and is widespread among eukaryotic mRNAs (Zhang et al., 2019). The key functions of m^7^G include protecting RNA from nuclease degradation, enhancing its stability, and facilitating RNA translation and nuclear export (Merrick and Pavitt, 2018).

Emerging research increasingly suggests the significance of m^7^G modification in AD, particularly in relation to neurogenesis, immune regulation, and the classification of AD subtypes (Ma et al., 2023). The m^7^G modification also facilitates the generation and maturation of neurons. Specifically, the Mettl1-driven m^7^G modification enhances the stability and translational efficiency of Sptbn2 mRNA, promoting neuronal development. The Mettl1-driven m^7^G modification may mitigate neurodegenerative damage in AD by fostering neuron generation and improving synaptic function (Li et al., 2023a). Additionally, m^7^G modification is closely associated with immune features in AD, as it regulates immune-related genes and modulates the intensity and duration of inflammatory responses (Lian et al., 2024).

### Uracil

The metabolic dysregulation of uracil, a key component of RNA, has been linked to neuronal aging and neurodegenerative diseases. In adult neurons, DNA polymerase β repairs DNA damage by regulating dUTP concentration. However, as neurons cease to proliferate, uracil DNA glycosylase activity decreases, leading to the accumulation of uracil in DNA and resulting in genomic instability (Mazzarello et al., 1990). Uracil concentrations are significantly reduced in AD, indicating nucleic acid metabolism disturbances (Ozaki et al., 2022). Additionally, folate deficiency increases the misincorporation of uracil into DNA, triggering DNA damage and enhancing susceptibility to Aβ peptide toxicity (Semenov et al., 2022).

### Pseudouridine

Pseudouridine modification in RNA involves both RNA-dependent and RNA-independent mechanisms. RNA-dependent pseudouridine synthetases, such as dyskerin, modify rRNA by binding to small nucleolar RNAs. In contrast, RNA-independent pseudouridine synthetases (e.g., PUS1–PUS7) directly recognize RNA sequences and structures to catalyze modifications (Hamma and Ferré-D’Amaré, 2006; Carlile et al., 2014; Lovejoy et al., 2014). Pseudouridine modifications are found in rRNA, tRNA, and mRNA, where they can alter RNA secondary structure, influence base-pairing properties, and even affect translation termination (Davis, 1995; Fernández et al., 2013; Kierzek et al., 2014). While elevated pseudouridine levels have been detected in the urine of patients with neurodegenerative diseases, such as AD, no direct pathological symptoms have been linked to this observation (Lee et al., 2007). A previous study has indicated that the deletion of PUS3 affects not only RNA modification but also neural development and function (Lin et al., 2022). Additionally, high expression levels of dyskerin 1, the pseudouridine synthetase component of the H/ACA-box snoRNP complex, have been observed in mouse embryonic neural tissues and specific neurons of the cerebellum and olfactory bulb in adult mice. This suggests that it may play a role in neural development (Heiss et al., 2000). Thus, abnormal pseudouridine modification may contribute to the onset and progression of neurodegenerative diseases by disrupting RNA stability and function, thereby affecting neuronal metabolism. However, the precise underlying mechanisms remain to be fully elucidated.

## Cell-Specific Regulation of m^6^A Modification in Neurodegenerative Diseases

The role of m^6^A modification in neurodegenerative diseases extends beyond general RNA regulation to produce distinct effects that depend on the specific neural cell type involved. Variations in m^6^A modification across different cell types can influence disease onset and progression through various mechanisms (Shi et al., 2019; Huang et al., 2020a). This section explores m^6^A modification changes to microglia, astrocytes, neurons, and adult neural stem cells in AD, PD, MS, and ALS (**Additional Table 3**).

**Additional Table 3:** Cell-specific regulation of m^6^A modification in neurodegenerative diseases

Cell type	Disease	Role	Reference
Microglia	AD	METTL3 promotes the release of inflammatory cytokines, exacerbating neuroinflammation. FTO regulates inflammatory responses and microglial migration, with its knockdown exacerbating both inflammation and microglial movement. m^6^A influences microglial polarization by promoting Ml polarization, which enhances pro-inflammatory cytokine release, while inhibiting M2 polarization reduces anti-inflammatory effects. Changes in METTL3 and ALKBH5 lead to microglial dysfunction, which reduces the clearance of Aβ and the degradation of Tau, thereby accelerating disease progression.	Wen et al., 2022; He et al., 2023b; Li et al., 2023b; Jiang et al., 2024
Astrocytes	AD	Increased levels of METTL3 and decreased levels of ALKBH5 suggest that m^6^A modification may play a role in regulating astrocyte function. The upregulation of FTO and YTHDF1 indicates that m^6^A modification is associated with disease progression. Additionally, the FTO inhibitor MO-I-500 significantly reduces astrocyte damage and regulates bioenergetic metabolism.	Cockova et al., 2021; Jiang et al., 2024
	PD	Downregulation of YTHDF2 enhances the pro-inflammatory response, while its overexpression inhibits this effect. YTHDF2 regulates inflammation by binding to the mRNA of MAP2K4 (MKK4), thereby activating the SEKl-JNK-cJUN signaling pathway.	Malovic et al., 2024
Neurons	AD	METTL3 upregulation and ALKBH5 downregulation affect neuronal function. Microglia activate PTPRG through the Mic_PTPRG subpopulation, leading to the upregulation of VIRMA, inhibition of PRKN translation, impairment of mitochondrial clearance, and promotion of neuronal death. METTL3 overexpression alleviates Aβ-induced synaptic damage and cognitive impairment. METTL14 stabilizes CBLN4 mRNA, thereby reducing Aβ-induced apoptosis and oxidative stress. Downregulation of ALKBH5 increases m^6^A modification, contributing to neuroinflammation and neuronal death.	Du et al., 2020; Wang et al., 2020; Zhao et al., 2021; Jiang et al., 2023b; Zou et al., 2024
	PD	The upregulation of ALKBH5 and IGF2BP2, along with the downregulation of YTHDF1 and FMR1, is associated with neuronal loss and motor defects in PD. Reduced m^6^A levels lead to increased expression of NMDA receptors, which heightens oxidative stress and calcium influx, ultimately exacerbating apoptosis in dopaminergic neurons. FTO knockout alleviates neuronal death by inhibiting α-syn upregulation and TH downregulation. METTL3 stabilizes GLRX mRNA, thereby slowing disease progression and reducing neuronal degeneration. Salsolinol exposure increases m^6^A levels, exacerbating dopaminergic neuron death by regulating YAP1 and autophagy.	Chen et al., 2019b; Yu et al., 2022; Geng et al., 2023; Gong et al., 2024; Wang et al., 2025
	ALS	RBMX mutations in ALS lead to motor neuron death and activate p53. Genes regulated by RBMX affect excitatory neurons, highlighting the role of m^6^A in ALS progression.	He et al., 2024b
Adult NSCs	AD, PD, ALS, MS	Loss of FTO promotes initial proliferation but disrupts neurogenesis, while loss of METTL3 inhibits NSC proliferation and skews differentiation toward glial cells. YTHDF2 maintains NSC quiescence and regulates neurogenesis through the TGF-β pathway. m^6^A modification also influences neuronal development by regulating genes such as *Lrp2* and *SLIT2.*	Chen et al., 2019a; Cao et al., 2020; Xu et al., 2022b; Zhao et al., 2023; Zhang et al., 2025

AD: Alzheimer's disease; ALKBH5: alkB homolog 5; ALS: amyotrophic lateral sclerosis; Aβ: amyloid-beta; CBLN4: Cerebellin 4; ceRNA: competing endogenous RNA; FMR1: fragile X messenger ribonucleoprotein 1; FTO: fat mass and obesity-associated protein; GLRX: glutaredoxin; IGF2BP2: insulin-like growth factor 2 mRNA-binding protein 2; IL-1β: interleukin-1 beta; JNK: c-Jun N-terminal kinase; Lrp2: low-density lipoprotein receptor-related protein 2; MAP2K4 (MKK4): mitogen-activated protein kinase kinase 4; METTL3: methyltransferase–like 3; METTL14: methyltransferase-like 14; m^6^A: N^6^-methyladenosine; MS: multiple sclerosis; NMDA: N-methyl-D-aspartate; NSC: neural stem cells; PD: Parkinson's disease; PRKN: Parkin RBR E3 ubiquitin protein ligase; PTPRG: protein tyrosine phosphatase receptor type G; RBMX: RNA-binding motif protein X; SEK1: SAPK/ERK kinase 1; SLIT2: slit guidance ligand 2; TGF-3: transforming growth factor-beta; TH: tyrosine hydroxylase; TNF-α: tumor necrosis factor-alpha; VIRMA: vir-like m^6^A methyltransferase-associated protein; YAP1: yes-associated protein 1; YTHDF1: YTH domain-containing family protein 1; YTHDF2: YTH domain-containing family protein 2.

### N^6^-Methyladenosine modification in microglia

Microglia, the primary immune cells of the CNS, originate from embryonic hematopoietic stem cells and are widely distributed throughout the brain and spinal cord (Pont-Lezica et al., 2011; Tay et al., 2019). They are vital for nervous system development, immune surveillance, and injury repair (Yuan et al., 2019). In neurodegenerative diseases, such as AD (Hansen et al., 2018), PD (Isik et al., 2023), and MS (Hammond et al., 2024), microglial function and state undergo significant alterations.

Microglial activation is triggered by the deposition of Aβ, representing a hallmark of early AD. Activated microglia exacerbate the neuroinflammatory response by releasing pro-inflammatory cytokines such as TNF-α, IL-1β, and IL-6 (AmeliMojarad and AmeliMojarad, 2024). These factors contribute to neuronal damage and accelerate neurodegeneration. Chronic microglial activation leads to a “vicious cycle,” whereby the sustained release of inflammatory factors triggers further microglial activation and worsens neuronal injury (Xu et al., 2022d). In lipopolysaccharide (LPS)-mediated microgliosis, METTL3 expression is increased, promoting the secretion of inflammatory cytokines (such as IL-1β, IL-6, TNF-α, and IL-18) and upregulating inflammation-related proteins (such as TRAF6 and NF-κB). METTL3 binds to TRAF6 and promotes the activation of the TRAF6-NF-κB signaling pathway in an m^6^A-dependent manner, intensifying the microglial inflammatory response (Wen et al., 2022). Reduced expression of FTO and its knockdown exacerbated the secretion of inflammatory factors, as well as microglial mobility and chemotaxis, in a uveitis model. FTO regulates GPC4 expression, thereby influencing the TLR4/NF-κB signaling pathway and enhancing the inflammatory response in microglia (He et al., 2023b). Additionally, m^6^A modification can influence microglial polarization by regulating signaling pathways and transcription factors. For instance, m^6^A modification can promote M1 polarization, enhancing the release of pro-inflammatory cytokines, while inhibiting M2 polarization reduces anti-inflammatory effects (Li et al., 2023b). Thus, m^6^A modifications are essential in determining the functional state of microglia and directly shape the inflammatory environment in the brain (Zhang et al., 2022).

Beyond Aβ accumulation, the aberrant phosphorylation and aggregation of tau proteins are central pathological features of AD (Bakota and Brandt, 2016). Microglia contribute to tau degradation by phagocytosing tau aggregates, thereby reducing neuronal damage associated with tau pathology. However, microglial dysfunction in AD hinders the effective clearance of tau proteins (Ayyubova, 2023). Furthermore, microglia modulate tau degradation pathways through m^6^A modification, including the involvement of METTL3, which may indirectly influence tau metabolism. In AD mouse models, the accumulation of Aβ plaques is accompanied by pathological tau aggregation, significant inflammation, and extensive neurodegeneration. m^6^A modification (involving METTL3 and ALKBH5) increases significantly during this process, primarily in the co-localization of neurons, microglia, and astrocyte subpopulations. This suggests that m^6^A may regulate tau function and degradation through interactions between microglia and other cell types (Jiang et al., 2024). Microglia also play a critical role in the neuroinflammatory response in PD via a similar mechanism. As the disease progresses and dopaminergic neurons are lost, microglia become activated and release large amounts of pro-inflammatory factors, such as IL-1β, TNF-α, and nitric oxide (Członkowska et al., 1996; Williams-Gray et al., 2016). This release worsens neuronal damage and cell death (George et al., 2019). Conversely, microglia can establish connections with neurons through tunneling nanotubes, facilitating the rapid exchange of organelles, vesicles, and proteins, which helps maintain neuronal health. This process alleviates oxidative stress, normalizes gene expression, and exerts a neuroprotective effect (Scheiblich et al., 2024). The direct relationship between m^6^A and microglia in PD and ALS remains largely unexplored at present.

### N^6^-Methyladenosine modification in astrocytes

Astrocytes, the predominant glial cells in the CNS, are vital for maintaining neural homeostasis and supporting neuronal function. Recent studies have explored the role of m^6^A modification in astrocytes, particularly in neurodegenerative diseases such as AD. Research indicates that there is a significant accumulation of m^6^A modifications in neurons, astrocytes, and microglia in AD model mice, alongside increased METTL3 expression and decreased ALKBH5. This suggests a potential regulatory role of m^6^A modification in astrocytes, indicating that these modifications may influence astrocytic function and, consequently, neuronal health (Jiang et al., 2024). Furthermore, studies involving human astrocyte tumors (specifically, CCF-STTG1) have altered m^6^A modification patterns under the AD streptozotocin model. Notably, the expression levels of the m^6^A demethylase FTO and the m6A reader YTHDF1 were significantly increased. The FTO inhibitor MO-I-500 demonstrated a protective effect against astrocyte damage induced by streptozotocin treatment, including reductions in oxidative stress and cell apoptosis, and increased glial fibrillary acidic protein expression, as well as improvements in mitochondrial function and overall bioenergetic stability (Cockova et al., 2021). These findings indicate the significance of the m^6^A signaling pathway in regulating astrocytic metabolism and its potential contribution to the progression of neurodegenerative diseases. As such, m^6^A modification alterations in astrocytes may emerge as critical regulatory mechanisms in these diseases, offering new avenues for therapeutic interventions. Further research is warranted to fully elucidate the implications of m6A modifications in astrocytes and their broader impact on CNS health and disease.

Astrocytes are crucial in PD. Beyond supporting neuronal function, they contribute to neurodegeneration through reactive proliferation and pro-inflammatory responses (Sofroniew, 2009; Phatnani and Maniatis, 2015; Patani et al., 2023). Additionally, interactions between astrocytes and microglia promote PD progression (Miyazaki and Asanuma, 2020). However, the role of astrocytes in PD is not entirely negative. In certain contexts, astrocytes can alleviate neurodegeneration and reduce dopaminergic neuron loss by secreting neuroprotective molecules such as GLP1R and NURR1 (Zhu et al., 2023). While there are few direct studies examining the impact of m^6^A modification in astrocytes on PD progression, existing research suggests that the neurotoxic stressor manganese (Mn) downregulates YTHDF2, thereby enhancing the inflammatory response in astrocytes. Knockdown of YTHDF2 exacerbated the pro-inflammatory response, while its overexpression inhibited this effect. YTHDF2 regulates inflammation by binding to MAP2K4 (MKK4) mRNA, activating the SEK1-JNK-cJUN signaling pathway (Malovic et al., 2024). Therefore, we speculate that, in PD, m^6^A modification may contribute to the inflammatory response and exacerbate neurodegeneration by regulating YTHDF2 in astrocytes.

The blood–brain barrier (BBB) is compromised in MS, allowing peripheral immune cells to invade the lesion area and activate astrocytes (Ortiz et al., 2014; Niu et al., 2019). In turn, reactive astrocytes exacerbate the inflammatory response by secreting chemokines (e.g., CCL2 and CXCL10) that promote leukocyte infiltration (Sanmarco et al., 2021; Lee et al., 2022). T cells regulate astrocyte activity by secreting cytokines (e.g., IL-10, GM-CSF) that influence both pro- and anti-inflammatory responses (Mayo et al., 2016). Additionally, environmental factors, metabolic intermediates, and the gut microbiota influence MS progression by activating astrocytes via the aryl hydrocarbon receptor (AHR) (Rothhammer et al., 2016; Gutiérrez-Vázquez and Quintana, 2018; Linnerbauer et al., 2023). The unfolded protein response signaling pathway also plays a critical role in MS, influencing its progression through astrocyte activation (Stone and Lin, 2015; Hetz et al., 2020). In experimental autoimmune encephalomyelitis, a model of MS, astrocytes exhibited reduced NRF2 and increased MAFG expression, with the latter cooperating with MAT2α to suppress antioxidant and anti-inflammatory transcription programs, promoting pro-inflammatory responses (Liddelow et al., 2017).

In ALS mice, single-nucleus RNA sequencing (snRNA-seq) analysis revealed neurotoxic, subtype-specific transcriptomic abnormalities in astrocytes. Restoring miR-146a in ALS astrocytes reversed the neurotoxic phenotype, enhancing lysosomal activity and increasing synaptic and axonal gene expression in motor neurons, suggesting that modulating miR-146a could restore normal astrocyte function and improve motor neuron dynamics in ALS (Gomes et al., 2022). Astrocyte activation has been closely linked to impaired glutamate recycling, aggregation of the mutant protein TDP-43, and neuroinflammation (Kwon and Koh, 2020; Smethurst et al., 2020; Westi et al., 2023). Bone morphogenetic protein 4 has been identified as a key factor promoting astrocyte generation and activation, with its expression gradually upregulated in ALS models (Shijo et al., 2018). As well as their neurotoxic roles, astrocytes also have protective functions, maintaining neuronal health by inhibiting TDP-43 aggregation and secreting neurotrophic and immunological factors (Kim et al., 2021). The dual neurotoxic and protective roles of astrocytes in ALS involve multiple mechanisms, including cytokine regulation (e.g., IL-6, TGF-β, IFN-γ) and mitochondrial dysfunction (Cassina et al., 2008; Hashioka et al., 2009; Shibata et al., 2009; Phatnani and Maniatis, 2015). Research on m6A modification in ALS and MS is relatively limited. However, m^6^A has been shown to play a regulatory role in astrocytes, such as by modulating xCT and related glutamate levels, greatly affecting neuronal excitability and neuroinflammatory responses (Zhang et al., 2024a). Given that astrocytes play a crucial role in the pathogenesis of both ALS and MS, m6A’s potential effect on astrocyte function, while not fully understood, offers new insights and may provide valuable therapeutic directions for both diseases.

### N^6^-Methyladenosine modification in neurons

Neurons are the core units of the nervous system, responsible for transmitting and processing information and forming neural networks to support perception, motor control, and memory functions (Lovinger, 2008). In neurodegenerative diseases such as AD, PD, and ALS, neurons’ structure and function deteriorate, resulting in cognitive impairments and motor deficits (Ransohoff, 2016).

METTL3 expression is increased in AD, while ALKBH5 expression is decreased in the neurons of AD model mice (Jiang et al., 2023b). Microglia interact with neurons via the microglial PTPRG subpopulation, which induces the expression of PTPRG. This, in turn, upregulates the m^6^A methyltransferase VIRMA, inhibiting PRKN mRNA translation, impairing mitochondrial clearance, and promoting neuronal death (Zou et al., 2024).

Additionally, reduced expression of the m^6^A methyltransferase METTL3 leads to decreased m^6^A modification in hippocampal neurons, triggering memory deficits, synaptic loss, and neuronal death. However, METTL3 overexpression can alleviate Aβ-induced synaptic damage and cognitive impairment (Zhao et al., 2021). Furthermore, METTL14 has been shown to reduce Aβ-induced apoptosis and oxidative stress, thereby decreasing AD-related neuronal damage by stabilizing CBLN4 mRNA (Mu et al., 2024). ALKBH5 is crucial for maintaining nervous system health and development, but its expression decreases during brain maturation, a process closely associated with functional refinement (Wang et al., 2020). Reduced ALKBH5 expression may lead to elevated m^6^A modification levels, affecting neuronal stability and activity. The diminished expression of ALKBH5 in AD could contribute to increased m^6^A levels, influencing key pathological mechanisms such as neuroinflammation, synaptic loss, and neuronal death (Du et al., 2020).

The most striking pathological feature of PD is the severe loss of dopamine neurons in the substantia nigra (SN), leading to abnormalities in the neural circuits responsible for motor control. This degeneration results in symptoms such as stiffness and resting tremors. Research has shown that at least 50% of neurons in the SN degenerate before a clinical diagnosis can be made (Ross et al., 2004). Additionally, α-synuclein (α-syn), the protein implicated in PD, accumulates in nigrostriatal neurons, forming Lewy bodies. This accumulation may trigger neuronal death through mechanisms such as mitochondrial dysfunction and oxidative stress (Tanaka et al., 2001; Yasuda et al., 2013).

A growing body of PD research highlights the critical role of m^6^A modification in the death of dopaminergic neurons. One study demonstrated that the upregulation of ALKBH5 and IGF2BP2, along with the downregulation of YTHDF1 and FMR1 in the SN and striatum, were closely linked to neuronal loss and motor defects in an MPTP-treated mouse model (Yu et al., 2022). Another study found that a reduction in m^6^A increased the expression of N-methyl-D-aspartate receptors, which in turn heightened oxidative stress and calcium influx, exacerbating dopaminergic neuron apoptosis (Chen et al., 2019b). Additionally, knockout of FTO was shown to inhibit the upregulation of α-syn and the downregulation of tyrosine hydroxylase, alleviating neuronal death in a PD model (Geng et al., 2023). Another investigation revealed an association between decreased expression of GLRX, a regulator of dopaminergic neuron death, and motor dysfunction and neuronal degeneration in PD. METTL3 could slow disease progression by stabilizing the m^6^A modification of GLRX mRNA (Gong et al., 2024). Furthermore, research has found that salsolinol, a neurotoxic environmental toxin linked to PD, exacerbated neurotoxicity by affecting m^6^A RNA methylation. Exposure to salsolinol increased m^6^A modification levels in PC12 cells, exacerbating dopaminergic neuron death by regulating YAP1 and autophagy (Wang et al., 2025).

ALS primarily affects upper and lower motor neurons, resulting in symptoms in the limbs and cranial nerves. ALS pathology is characterized by the cytoplasmic aggregation of TDP-43, which is strongly associated with neuronal loss (Vance et al., 2009; Brettschneider et al., 2014). The abnormal accumulation of TDP-43 not only leads to mitochondrial dysfunction but also binds to mitochondrial mRNA, inhibiting the expression of respiratory complex I and causing its disassembly (Tsuiji et al., 2017; Davis et al., 2018). Although evidence establishing whether apoptosis is the primary pathway for neuronal death remains inconclusive, TDP-43 may induce neuronal death via the p53 pathway (Vogt et al., 2018). The role of m^6^A modification in ALS is unclear, but mutations in m^6^A-related genes, especially in the RNA-binding motif protein X-linked (RBMX), are linked to the disease. Whole-exome sequencing revealed that RBMX variants were enriched in ALS patients and associated with poor clinical outcomes. In ALS motor neurons, RBMX knockdown led to cell death and p53 activation, suggesting that m^6^A modification plays a role in neuronal dysfunction. Transcriptomic analysis revealed that RBMX-regulated genes specifically affect excitatory neurons and are enriched in ALS-related pathways, highlighting the potential role of m^6^A in ALS progression (He et al., 2024b).

### N^6^-Methyladenosine modification in adult neural stem cells

NSCs are one of the few types of stem cells capable of differentiating into neural cells in the adult brain, and are primarily found in regions such as the olfactory bulb, hippocampus, and lateral ventricular zone (Beltz et al., 2015; Zhao and Moore, 2018). Possessing self-renewal capabilities, NSCs can differentiate into various cell types, including neurons and glial cells (e.g., astrocytes and oligodendrocytes), depending on physiological and pathological conditions (Corvaglia et al., 2019; Liu et al., 2023a). These cells are crucial for neural development, neuroplasticity, and brain repair. Maintaining their normal function is essential for cognitive processes such as learning and memory (Mao and Wang, 2003; Dietrich and Kempermann, 2006) and for the treatment of various neurodegenerative diseases, including AD (Boese et al., 2020; Han et al., 2020a; Walgrave et al., 2021), PD (Storch and Schwarz, 2002; Pardal and López-Barneo, 2012; Li et al., 2022a), MS (Peruzzotti-Jametti et al., 2021; Genchi et al., 2023; Leone et al., 2023), and ALS (Mitrecić et al., 2009; Mazzini et al., 2019). Research has primarily focused on NSC transplantation and treatment under these conditions.

Research on m^6^A modification in NSCs has increased significantly in recent years. One study found that the loss of FTO in adult NSCs initially promoted cell proliferation and neuronal differentiation; however, it inhibited adult neurogenesis and neuronal development in the long term (Cao et al., 2020). The loss of FTO disrupted the normal neurogenic process by altering the m^6^A modification of Pdgfra and Socs5, leading to Stat3 phosphorylation. Additionally, the loss of METTL3 inhibited NSC proliferation and skewed neuronal differentiation toward glial cell types, while also affecting the morphological maturation of newborn neurons. METTL3 influenced neurogenesis and neuronal development by regulating Ezh2 expression. Overexpression of Ezh2 could partially rescue the neurogenic defects caused by METTL3 loss (Zhao et al., 2023). Another study revealed that YTHDF2, an m^6^A reader protein, plays a crucial role in maintaining NSC quiescence. Its loss promoted NSC activation and reduced neurogenesis through the TGF-β signaling pathway (Zhang et al., 2025a). Furthermore, m^6^A modification has been reported to regulate the expression of genes essential for neurogenesis, such as *Lrp2* and *SLIT2*. Overexpression of these genes could partially rescue neuronal development defects caused by unbalanced m^6^A regulation (Chen et al., 2019a; Xu et al., 2022b). Therefore, m^6^A modification is a vital mechanism for regulating the development and function of neural stem cells; this provides important insights into neurogenesis and its potential role in neurodegenerative diseases.

## Therapeutic Potential of Targeted N^6^-Methyladenosine Modification in Neurodegenerative Diseases

### Therapeutic potential of methyltransferase-like 3

METTL3, a key element of the m^6^A methyltransferase complex, adds methyl groups to adenosine residues in RNA, thereby generating m^6^A modification. As the central enzyme in this process, METTL3 is crucial for numerous biological functions, such as regulating gene expression, determining cell fate, embryogenesis, neural development, and maintaining nervous system functionality (Liu et al., 2020).

In autopsy samples of human brain tissue, METTL3 expression changed significantly, particularly in the hippocampus, where the expression of the METTL3-encoding gene decreased while the expression of the RBM15B gene was upregulated (Huang et al., 2020b). Additionally, METTL3 expression was closely correlated with Mini-Mental State Examination scores, with low METTL3 levels associated with a higher risk of AD. This suggests a potential role for METTL3 in mRNA protection and AD prevention (Yang et al., 2023). Reduced METTL3 levels led to oxidative stress and neuronal damage, while restoring m^6^A modifications or overexpressing METTL3 was found to alleviate AD symptoms (Zhao et al., 2021). Additionally, METTL3 deficiency improved Aβ-induced cognitive dysfunction by regulating m^6^A modification, enhancing Aβ clearance, and alleviating associated symptoms (Yin et al., 2023). Furthermore, METTL3 enhanced MFN2 expression through m^6^A modification, improving mitochondrial dysfunction in an AD mouse model. Overexpression of either METTL3 or MFN2 could mitigate cognitive impairment and enhance mitochondrial function, further indicating the potential of METTL3 in treating AD (Chen et al., 2024). In summary, METTL3 may act as a key factor in the pathogenesis of AD through its involvement in various mechanisms.

METTL3 mRNA levels in patients with PD were found to be significantly lower than those in healthy controls (Yu et al., 2022; He et al., 2023a). Notably, NRF1 has been shown to regulate METTL3 expression, which enhances the m^6^A modification of GLRX mRNA. This modification alleviates dopamine neuron degeneration, improves motor dysfunction, and reduces neurodegenerative lesions in PD mouse models (Gong et al., 2024). In contrast, an increase in METTL3 expression has been observed in patients with MS, suggesting a potential link to the neurodegenerative changes associated with this condition (Ye et al., 2021).

In ALS, METTL3 plays a dual role in regulating the aggregation and degradation of FUS through m^6^A modification. Research has demonstrated elevated m^6^A levels in neurons with mutant FUS, which correlated with increased FUS aggregate formation in induced pluripotent stem cell–derived motor neurons. Reducing m^6^A levels decreased FUS inclusions, making them vulnerable to lysosomal degradation. The METTL3 inhibitor STM-2457 also reduced FUS inclusions, supporting the idea that METTL3, through m^6^A modification, might influence FUS aggregation in ALS, offering a potential therapeutic approach (Di Timoteo et al., 2024). Furthermore, it has been found that changes in METTL3 were related to the regulation of HSV-1 virus infection. HSV-1 infection enhanced METTL3 expression, particularly during the early stages of infection. Inhibiting m^6^A modification or silencing METTL3 with siRNA significantly reduced viral replication and reproduction, indicating the significant involvement of METTL3 in viral replication (Feng et al., 2022). Changes in METTL3 expression may influence the occurrence and progression of viral infections in ALS.

### Therapeutic potential of fat mass and obesity–associated protein

FTO is a key m^6^A demethylase involved in erasing m^6^A modification from RNA molecules (Wei et al., 2018). FTO influences RNA stability and translational efficiency by erasing m^6^A modification (Berulava et al., 2020). FTO is thought to contribute to various neurodegenerative diseases due to its central role in regulating m^6^A modification (Shu et al., 2021).

In the dynamic equilibrium of m^6^A modification, FTO acts as an ‘eraser’ to regulate AD-related gene expression in neurons. The NIA-LOAD study found that FTO expression in AD was decreased in comparison with controls (Reitz et al., 2012). Genetic variations in FTO interacted with APOE alleles, heightening susceptibility to AD (Keller et al., 2011). FTO also contributed to neurodegeneration by affecting the TSC1-mTOR-Tau signaling pathway, which promotes abnormal accumulation of tau proteins (Li et al., 2018). Additionally, FTO contributed to adult neurogenesis and hippocampal memory processes, affecting cognitive function as well as NSC growth and specialization through the regulation of adenosine metabolism and the lipid microenvironment (Gao et al., 2020). FTO has also been shown to mitigate Aβ_1–40_-induced degeneration in retinal pigment epithelial cells via activation of the PKA/CREB pathway, which plays a role in neuronal damage caused by Aβ (Chen et al., 2012; Hu et al., 2023). Overall, FTO is integral to the complex pathological processes of AD and represents a promising therapeutic target.

Overexpression of FTO has been shown to reduce m^6^A levels, mitigate manganese-induced cytotoxicity, and alleviate PD symptoms (Qi et al., 2022). This study demonstrated that FTO inhibitors promoted the survival of dopaminergic neurons in the mouse brain, effectively rescuing them from growth factor deprivation-induced apoptosis. Notably, these inhibitors exhibited significant protective effects on dopaminergic neurons at nanomolar concentrations. Furthermore, they demonstrated good permeability across the BBB, indicating their potential for neuroprotection and treatment of neurodegenerative diseases (Selberg et al., 2021). FTO also demethylated D2R and D3R receptors, which are involved in dopaminergic transmission, learning, and reward behavior, thereby contributing to neurological dysfunction in PD (Hess et al., 2013). Although direct studies on the dopaminergic role of FTO in PD are limited, these findings suggest its potential involvement in the disease, particularly in regulating dopaminergic transmission and influencing neurological dysfunction. Additionally, FTO overexpression increased apoptosis in PC12 cells and exacerbated PD pathology by enhancing mitochondrial production of reactive oxygen species (Chen et al., 2019b). Collectively, these findings suggest that FTO plays a key role in the pathogenesis of PD; therefore, targeting FTO may offer new therapeutic strategies for treating the disease.

*FTO* gene variants have been positively associated with sporadic amyotrophic lateral sclerosis in Greek patients; however, similar associations have not been observed in other populations, possibly due to a founder effect in Greece. The high FTO expression in motor neurons suggests its involvement in the genetic mechanisms underlying ALS (Mitropoulos et al., 2017).

Polymorphisms in the *FTO* gene, particularly rs9939609, have been linked to obesity and disability in patients with MS, although they do not appear to influence the risk of developing MS. The A allele of this polymorphism is associated with increased risk of obesity and disability. Additionally, FTO has been shown to influence homocysteine levels in MS, indicating its involvement in metabolic processes related to the disease. These findings indicate the role of FTO in epigenetic regulation and the effect of diet on the health of individuals with MS (Davis et al., 2014; Al-Serri et al., 2019).

### Therapeutic potential of AlkB homolog 5

AlkB homolog 5 (ALKBH5), an RNA demethylase, plays an essential role in regulating RNA modification, particularly in removing m^6^A marks. ALKBH5 specifically catalyzes the removal of the m^6^A modification, affecting RNA stability, splicing, and translation (Zheng et al., 2013). This process is crucial for biological functions, including gene expression, RNA processing, and cellular responses to stress. As an m^6^A eraser, ALKBH5 is associated with several human diseases, including neurological disorders, cancers, and metabolic conditions (Wang et al., 2020; Sun et al., 2023; Kuang et al., 2024; Su et al., 2024). The reduced expression of ALKBH5 in AD has been established (Jiang et al., 2023a); in contrast, it has been found to be upregulated in the substantia nigra in PD (Qiu et al., 2020; Yu et al., 2022).

ALKBH5 also regulates the expression of heme oxygenase 1 (HO-1), an enzyme that plays a crucial role in antioxidant defense and cellular protection (Ryter, 2021). By removing m^6^A modifications from HO-1, ALKBH5 helps to regulate ferroptosis, which is an iron-dependent form of cell death implicated in several neurodegenerative diseases (Yan et al., 2021). In experimental studies, inhibiting ALKBH5 activity resulted in a reduction of HO-1 expression, thereby promoting ferroptosis. Conversely, overexpression of ALKBH5 increased HO-1 levels, protecting neurons from cobalt-induced injury by inhibiting ferroptosis. These findings position ALKBH5 as a promising candidate for addressing neurodegenerative conditions related to ferroptosis and iron-dependent cell death (Su et al., 2024).

### Therapeutic potential of YTH N^6^-methyladenosine RNA-binding proteins

YTHDF1 and YTHDF2 are essential m^6^A reader proteins that play significant roles in various biological processes by recognizing m^6^A modifications on RNA. They influence RNA stability, translation efficiency, and localization (Wang et al., 2015). Specifically, YTHDF1 and YTHDF2 facilitate protein synthesis by enhancing the translation of m^6^A-modified mRNAs (Chen et al., 2023b).

Research indicates that YTHDF1 and YTHDF2 modulate the plasticity and adaptability of neural systems by regulating genes critical for synaptic activity and neuronal survival. YTHDF1 is believed to support synaptic function by promoting the translation of m^6^A-modified mRNAs (Shi et al., 2018). In the context of PD, YTHDF1 is downregulated in the SN (Yu et al., 2022). Additionally, YTHDF1 and YTHDF3 exhibit opposing expression patterns in the cerebellum, frontal lobe, and cingulate cortex (Martinez De La Cruz et al., 2023). Notably, YTHDF1 is involved in memory regulation, contributing to the storage of normal memory through the D1 (direct) pathway and inhibitory memory through the D2 (indirect) pathway. These findings suggest that YTHDF1 has potential therapeutic applications in PD, particularly in preserving normal memory and regulating motor memory (Wen et al., 2024). Moreover, studies have shown that exposure to single nucleotide polymorphisms enhances m^6^A methylation in the 5′ UTR of ferritin-linked protein catenin-acyl-CoA long-chain family member 4 (ACSL4) by recruiting the YTHDF1 protein, which amplifies ACSL4 expression and accelerates the ionization of dopaminergic neurons (Feng et al., 2024a). Additionally, YTHDF1 has been shown to enhance poly(GR) inclusion formation in models of ALS (Park et al., 2023). Furthermore, YTHDF2 has been implicated in modulating TDP43-mediated RNA binding in ALS, with knockout of YTHDF2 extending neuron survival, underscoring its critical role in the pathology of ALS (**Additional Table 4**).

**Additional Table 4:** Therapeutic potential of targeted m^6^A modifications in neurodegenerative diseases

m^6^A Regulator	Disease	Mechanism of action	Reference
METTL3	AD	METTL3 expression decreases in the hippocampus, with upregulation of RBM15B. Low METTL3 expression correlates with an increased risk of AD. METTL3 alleviates AD symptoms by restoring m^6^A modification, enhancing Aβ clearance, and improving mitochondrial function through the expression of MFN2.	Huang et al., 2020b; Zhao et al., 2021; Yang et al., 2023; Yin et al., 2023; Chen et al., 2024
	MS	METTL3 expression is increased in MS patients, which may be associated with the neurodegenerative changes observed in the disease.	Ye et al., 2021
	ALS	METTL3 regulates FUS aggregation and degradation via m^6^A modifications, and enhances HSV-1 replication. Reducing m^6^A or silencing METTL3 reduces FUS inclusions and viral replication, suggesting a role in ALS progression.	Feng et al., 2022; Di Timoteo et al., 2024
FTO	AD	FTO regulates the expression of AD-related genes in neurons. FTO expression is decreased in AD, and genetic variations in FTO interact with APOE alleles, increasing susceptibility to AD.	Keller et al., 2011; Chen et al., 2012; Reitz et al., 2012; Li et al., 2018; Gao et al., 2020; Hu et al., 2023
		FTO contributes to neurodegeneration via the TSC1-mTOR-Tau signaling pathway. It also affects cognitive function and neurogenesis, and mitigates Aβ_1-40_-induced damage through the PKA/CREB pathway.	
	PD	Overexpression of FTO reduces m^6^A levels and mitigates manganese-induced cytotoxicity. FTO inhibitors promote dopaminergic neuron survival and rescue from apoptosis.	Hess et al., 2013; Chen et al., 2019b; Selberg et al., 2021
		FTO demethylates D2R and D3R receptors, affecting dopaminergic transmission and contributing to neurological dysfunction. FTO also increases apoptosis in PC12 cells, exacerbating PD pathology.	
	ALS	FTO gene variants are associated with sporadic ALS in Greek patients, and its high expression in motor neurons suggests its involvement in ALS genetic mechanisms.	Mitropoulos et al., 2017
	MS	Polymorphisms in FTO, particularly rs9939609, are linked to obesity and disability in MS patients, but not MS risk. FTO also influences homocysteine levels, highlighting its role in MS metabolism and diet-related epigenetic regulation.	Davis et al., 2014; Al-Serri et al., 2019
ALKBH5	AD, PD, ALS, MS	ALKBH5 expression is reduced in AD and upregulated in the substantia nigra in PD. ALKBH5 regulates HO-1 expression, influencing ferroptosis, a process linked to neurodegenerative diseases. Inhibition of ALKBH5 promotes ferroptosis, while overexpression protects neurons by inhibiting ferroptosis. It also affects cognitive function and neurogenesis, and mitigates Aβ_1-40_-induced damage through the PKA/CREB pathway.	Qiu et al., 2020; Ryter, 2021; Yan et al., 2021; Yu et al., 2022; Jiang et al., 2023a; Su et al., 2024
YTHDF1 & YTHDF2	AD, PD, ALS, MS	YTHDF2 expression changes in AD, while YTHDF1 is downregulated in the substantia nigra in PD. YTHDF1 plays a role in memory regulation, preserving normal and inhibitory memory through the D1 and D2 pathways.	Yu et al., 2022; Martinez De La Cruz et al., 2023; McMillan et al., 2023; Park et al., 2023; Feng et al., 2024a;
		In ALS, YTHDF1 enhances poly(GR) inclusion formation and regulates TDP43-mediated RNA binding, extending neuron survival.	Wen et al., 2024

AD: Alzheimer's disease; ALKBH5: alkB homolog 5; ALS: amyotrophic lateral sclerosis; APOE: apolipoprotein E; Aβ_1-40_: amyloid-beta 1-40; CREB: cAMP response element-binding protein; D2R: dopamine D2 receptor; D3R: dopamine D3 receptor; FTO: fat mass and obesity-associated protein; FUS: fused in sarcoma; GLRX: glutaredoxin; HO-1: heme oxygenase-1; HSV-1: herpes simplex virus type 1; M^6^A: N^6^-methyladenosine; METTL3: methyltransferase-like 3; MFN2: mitofusin 2; MS: multiple sclerosis; mTOR: mechanistic target of rapamycin; NRF1: nuclear respiratory factor 1; PC12: pheochromocytoma cells 12; PD: Parkinson's disease; PKA: protein kinase A; poly(GR): glycine-arginine dipeptide repeat protein; RBM15B: RNA-binding motif protein 15B; TDP43: TAR DNA-binding protein 43; TSC1: tuberous sclerosis complex 1; YTHDF1: YTH domain-containing family protein 1; YTHDF2: YTH domain-containing family protein 2.

### Therapeutic potential of N⁶-methyladenosine regulatory axes

Beyond regulating individual m^6^A modulators, *in vivo* m^6^A methylation levels are more profoundly influenced by the synergistic interactions of multiple regulators, with the METTL3-YTHDF1 and FTO-YTHDF2 axes serving as two representative examples. In AD, Aβ has been shown to inhibit the m^6^A modification of ARC mRNA, leading to decreased ARC protein expression. Conversely, METTL3 overexpression enhances m^6^A modification, reversing this effect. YTHDF1, as an m^6^A ‘reader’ protein, plays a crucial role in this process, and its reduced expression significantly inhibits ARC expression. Therefore, the METTL3-YTHDF1 axis may serve as a potential therapeutic target for AD (Xu et al., 2022c).

Additionally, the FTO–YTHDF2 axis is critical in cobalt-induced neurodegenerative pathology. FTO destabilizes TSC1 mRNA and inhibits the TSC1/2-mTOR signaling pathway through an m^6^A-YTHDF2-dependent mechanism, leading to disrupted and impaired autophagic flux. This mechanism has been demonstrated in cobalt-exposed mice and hip replacement patients, suggesting that the FTO–YTHDF2 axis is also a potential therapeutic target for neurodegenerative injury (Tang et al., 2023a). Notably, these m^6^A modification axes are also significantly involved in other diseases, including metabolic and joint disorders (He et al., 2022; Xu et al., 2022a; Zhao et al., 2022; Wang et al., 2024b). Further in-depth studies on these pathways will provide novel therapeutic targets and directions for the application of epitranscriptomics in disease treatment.

## Clinical Translation Based on N^6^-methyladenosine Modification Regulation

There is currently a lack of effective cures for neurodegenerative diseases, including AD, PD, MS, and ALS, with existing treatments primarily focused on managing symptoms rather than addressing the underlying disease mechanisms. While treatments such as L-dopa for PD can provide symptom relief (LeWitt, 2015), they do not halt disease progression or reverse the loss of neuronal functions (Nutt, 2000; Demailly et al., 2024). Identifying new therapeutic targets that can slow or reverse disease progression remains a crucial focus of ongoing research. Recently, m^6^A RNA modification and its regulatory factors, such as METTL3, FTO, and ALKBH5, have emerged as promising candidates in the context of neurodegenerative diseases. m^6^A modifications are involved in regulating key processes such as RNA stability, gene expression, and cellular responses to stress. These modifications have a significant impact on neuronal health, synaptic plasticity, and cognitive functions, all of which are crucial for maintaining the nervous system’s integrity. Considering that m^6^A modifications play an important role in the pathophysiology of these diseases, the enzymes involved in this process represent a potentially effective therapeutic strategy (Suga et al., 2023). By modulating the activity of m^6^A-related enzymes, such as METTL3, FTO, and ALKBH5, researchers aim to slow the progression of neurodegenerative diseases like AD and PD, and potentially reverse neuronal damage.

### Clinical indications and potential targeted therapies of N^6^-methyladenosine modification in neurodegenerative diseases

METTL3, FTO, and ALKBH5 have emerged as promising targets for the treatment of these neurodegenerative diseases. Research on METTL3, particularly METTL3 inhibitors (e.g., STM2457), is primarily focused on its application in leukemia and other cancers (Liu et al., 2024), and it has demonstrated promising efficacy in basic research and animal models. STC-15 is the first METTL3 inhibitor to enter human clinical trials. According to Phase I clinical data presented by STORM Therapeutics at SITC 2024, STC-15 demonstrated good tolerability and significant tumor shrinkage across multiple cancer types, with sustained tumor reduction observed in three patients undergoing long-term maintenance therapy. STC-15 enhances the immune response by inhibiting METTL3, promoting M1-type macrophage enrichment, and strengthening anti-tumor immunity. The Phase II trial aims to further optimize the dosage and expand the indications, facilitating the clinical translation of m^6^A modification in cancer therapy (Wu et al., 2024d). However, the therapeutic potential of targeting METTL3 in neurodegenerative diseases remains unclear. While METTL3 activation may provide short-term benefits, its overactivation could result in undesirable side effects. Therefore, developing precise METTL3 modulators that restore neuronal function without adverse effects is crucial for future therapies. Although research on METTL3 agonists and inhibitors is still in its infancy (Yankova et al., 2021; Pan et al., 2023), particularly within basic research, a deeper understanding of m^6^A modification mechanisms and advances in drug development technologies suggest that METTL3-targeted therapies may become a breakthrough in the treatment of neurodegenerative diseases.

Similarly, FTO, a key m^6^A demethylase, has gained attention in relation to treatment for neurodegenerative diseases due to its association with disease progression. FTO inhibitors have demonstrated therapeutic potential, with FB23-2 significantly inhibiting tumor progression in AML xenograft models (Huang et al., 2019; Cockova et al., 2021). Currently, the FTO inhibitor bisantrene (CS1) is in clinical trials, undergoing a Phase II study (NCT03820908) in R/R AML. Among ten patients receiving CS1 (250 mg/m^2^, intravenously, for 7 days), the overall response rate was 40%, including one complete and three partial remissions. Given its low toxicity, future studies will explore CS1 in combination therapies (Canaani et al., 2021; Dong et al., 2025). FTO inhibitors also show promise for treating PD, with high-throughput screening identifying compounds that protect dopaminergic neurons, promote cell survival, and reduce apoptosis. These inhibitors effectively penetrate the BBB and may restore m^6^A modifications to slow neurodegeneration (Selberg et al., 2021). While FTO inhibitors have not yet been tested in AD, some inhibitors, such as methylamine, effectively restore m^6^A levels (Huang et al., 2015), highlighting FTO as a promising therapeutic target.

The development of ALKBH5 inhibitors has progressed through several stages, with candidate compounds initially identified via high-throughput screening and computer-assisted drug design. These inhibitors have since been optimized to enhance selectivity and inhibitory activity. Compounds such as IOX1 (Chen et al., 2023a), MV1035 (Malacrida et al., 2020), Ena15, and Ena21 (Takahashi et al., 2022) effectively inhibit ALKBH5 activity, affecting m^6^A modification and regulating the expression of related genes. These compounds have demonstrated therapeutic potential in models of cancer, acute kidney injury, and others, particularly in terms of inhibiting cell proliferation and promoting apoptosis, suggesting they could become novel therapeutic strategies (Wang et al., 2023d).

Through the continued research and development of these targeted drugs, new therapeutic options may emerge for patients with neurodegenerative diseases, especially AD and PD. Clinical trials are necessary to validate the safety and efficacy of these compounds and to lay the groundwork for personalized treatment strategies.

### Emerging technologies for studying N^6^-methyladenosine modification

Due to high complexity and cellular heterogeneity of the nervous system, the precise characterization of m^6^A functions across different cell types and specific brain regions remains elusive. Conventional bulk RNA sequencing (bulk RNA-seq) lacks the resolution to distinguish m^6^A regulatory patterns in distinct cell populations, thereby limiting a comprehensive understanding of its role in disease mechanisms. The emergence of single-cell RNA sequencing and spatial transcriptomics in recent years has provided powerful tools to elucidate the cell-type-specific functions of m^6^A modification in neurodegenerative disorders. These technologies enable a more refined analysis of m^6^A regulatory networks within specific cell populations and brain regions, offering novel insights into its role in disease pathogenesis.

#### Single-cell RNA sequencing

Recent studies integrating single-cell sequencing with m^6^A methylation analysis have begun to reveal its critical role in neurodegenerative diseases. For instance, single-cell m^6^A profiling of hippocampal tissues showed that m^6^A-modified transcripts were highly enriched in AD, particularly in CAMK2A-expressing neurons, suggesting that m^6^A modification may influence neuronal function and contribute to disease progression (Feng et al., 2024b). Similarly, in ALS, integrating single-cell transcriptomic data with m^6^A methylomics revealed aberrantly elevated m^6^A modification (permethylation) in peripheral immune cells of ALS patients (He et al., 2024a). These findings indicate the potential of combining single-cell sequencing with m^6^A methylation analysis to uncover cell type-specific regulatory mechanisms in neurodegenerative diseases, providing novel directions for precision medicine and targeted interventions.

#### Spatial transcriptomics

Spatial transcriptomic technologies have been increasingly applied in AD research in recent years, offering novel insights into disease-related spatial gene expression patterns. For instance, Stereo-seq has revealed laminar structural alterations and abnormal cell–cell interactions in the prefrontal cortex of AD patients (Gong et al., 2025). The integration of spatial transcriptomics technologies with m^6^A modification studies has further uncovered the role of RNA epigenetic regulation in AD. Spatial transcriptomic analyses of AD mouse models identified a specific microglia subpopulation (Mic_PTPRG) that interacted with neuronal subtypes via PTPRG to upregulate m^6^A methyltransferase VIRMA (Zou et al., 2024). Beyond elucidating AD-associated spatial transcriptional regulation, spatial transcriptomic technologies also provide insights into cell type-specific alterations to m^6^A modification.

### Clinical translational research challenges

While m^6^A modifications in therapeutic strategies hold great promise, the progression from research to clinical application involves several challenges, including:

#### Target selectivity

One of the major challenges is ensuring that therapeutic drugs specifically target relevant cell types, such as neuronsor glial cells (Su et al., 2020; Selberg et al., 2021; Li and Zhang, 2024). The behavior and regulatory mechanisms of these targets can differ significantly between cell types, making precise drug selectivity a crucial factor for achieving the intended effects in neurodegenerative diseases.

#### Drug delivery systems

The challenge is to ensure the effective passage of drugs through the BBB to reach the CNS. To address this issue, researchers are developing advanced drug delivery systems, such as nanoparticle carriers (Li et al., 2025) and liposomes (Wu et al., 2023), which can improve drug transport across the BBB and enhance treatment effectiveness.

#### Safety and side effects

A major concern in clinical trials is the potential side effects and long-term safety of novel drugs. Since m^6^A modifications affect a broad range of cellular processes in both neuronal and nonneuronal cells, it is crucial to minimize any unintended effects on healthy tissues while targeting neurodegenerative diseases. Ensuring the safety of treatments without disrupting normal cell function remains a significant challenge (Deng et al., 2022).

Therapeutic approaches focused on m^6^A modifications show considerable promise for clinical use in treating neurodegenerative diseases. Research on targets such as METTL3, FTO, and ALKBH5 has opened new avenues for treatment (Lv et al., 2023). However, further preclinical studies, improved trial designs, and advancements in drug delivery technologies are necessary to translate these targeted therapies into viable clinical options.

## Limitations

The limitations of this review primarily fall into the following areas: First, while the role of m^6^A methylation in various neurodegenerative diseases is summarized, current studies largely focus on basic research and animal models. There is a relative lack of clinical data, which prevents a comprehensive demonstration of the effectiveness of m^6^A modification in treating neurodegenerative diseases. Consequently, translating the findings of basic research into clinical treatments remains a significant challenge. Second, the specific mechanisms of m^6^A modification in different neural cell types have not been fully elucidated. Although this review discusses changes in m^6^A modification in microglia, astrocytes, neurons, and neural stem cells, the interactions between these cell types and their precise effects on disease progression require further investigation. Additionally, the synergistic effects between m^6^A modification and other RNA modifications (e.g., m^5^C, m^7^G) have not been adequately explored. Future studies will need to consider multiple modification mechanisms to fully understand the role of RNA modifications in neurodegenerative diseases. Finally, although m^6^A-modified regulators (e.g., METTL3, FTO, ALKBH5, YTHDF1) are promising therapeutic targets for neurodegenerative diseases, the clinical development of these targeted therapies is still in its early stages. Most of these drugs have only been validated in laboratory settings, and there is a lack of systematic clinical trial data. Therefore, further research and clinical trial data are needed to determine how therapeutic strategies targeting m^6^A modifications can be effectively applied in clinical practice. These limitations indicate that while m^6^A modifications hold great potential for neurodegenerative diseases, translating these findings into effective clinical therapies requires further in-depth research and exploration.

## Conclusions and Outlook

Although preliminary studies support the role of m^6^A modification in neurodegenerative diseases, its clinical translation still faces numerous challenges. The precise function of m^6^A modifications across various neurodegenerative diseases remains inadequately understood. Most current research primarily focuses on fundamental science and animal model testing, with limited exploration of human patient samples. Moreover, the variety of m^6^A regulators and the complex nature of their interactions present significant challenges in developing targeted therapies. While some m^6^A-related compounds have shown effectiveness in preclinical studies, their specificity, minimal toxicity, and sustained therapeutic effects in clinical applications still require comprehensive validation.

The advancement of gene editing technologies, precision medicine, and high-throughput screening methods is expected to facilitate the development of m^6^A-targeted therapies, potentially offering a groundbreaking approach to the treatment of neurodegenerative diseases. Multidisciplinary collaboration, particularly the integration of basic research and clinical application, will be crucial for advancing m^6^A modification research from the laboratory to the clinic.

## Additional files:

***Additional Table 1:*** *Roles of m*^*6*^*A modification in early and late stages of AD and AD animal models.*

***Additional Table 2:*** *Other RNA modifications in neurodegenerative diseases.*

***Additional Table 3:*** *Cell-specific regulation of m*^*6*^*A modification in neurodegenerative diseases.*

***Additional Table 4:*** *Therapeutic potential of targeted m*^*6*^*A modifications in neurodegenerative diseases.*

## Data Availability

*All relevant data are within the paper and its Additional files*.
